# Correlation between COVID-19 and weather variables: A meta-analysis

**DOI:** 10.1016/j.heliyon.2022.e10333

**Published:** 2022-08-18

**Authors:** Md. Momin Islam, Farha Musharrat Noor

**Affiliations:** a Department of Meteorology, University of Dhaka, Dhaka 1000, Bangladesh; b Department of Statistics, University of Dhaka, Dhaka 1000, Bangladesh

**Keywords:** COVID-19, Weather variables, Correlation, Meta-analysis

## Abstract

**Background:**

COVID-19 has significantly impacted humans worldwide in recent times. Weather variables have a remarkable effect on COVID-19 spread all over the universe.

**Objectives:**

The aim of this study was to find the correlation between weather variables with COVID-19 cases and COVID-19 deaths.

**Methods:**

Five electronic databases such as PubMed, Science Direct, Scopus, Ovid (Medline), and Ovid (Embase) were searched to conduct the literature survey from January 01, 2020, to February 03, 2022. Both fixed-effects and random-effects models were used to calculate pooled correlation and 95% confidence interval (CI) for both effect measures. Included studies heterogeneity was measured by Cochrane chi-square test statistic Q, $I^{2}$ and $\tau^{2}$. Funnel plot was used to measure publication bias. A Sensitivity analysis was also carried out.

**Results:**

Total 38 studies were analyzed in this study. The result of this analysis showed a significantly negative impact on COVID-19 fixed effect incidence and weather variables such as temperature (r = -0.113∗∗∗), relative humidity (r = -0.019∗∗∗), precipitation (r = -0.143∗∗∗), air pressure (r = -0.073∗), and sunlight (r = -0.277∗∗∗) and also found positive impact on wind speed (r = 0.076∗∗∗) and dew point (r = 0.115∗∗∗). From this analysis, significant negative impact was also found for COVID-19 fixed effect death and weather variables such as temperature (r = -0.094∗∗∗), wind speed (r = -0.048∗∗), rainfall (r = -0.158∗∗∗), sunlight (r = -0.271∗∗∗) and positive impact for relative humidity (r = 0.059∗∗∗).

**Conclusion:**

This meta-analysis disclosed significant correlations between weather and COVID-19 cases and deaths. The findings of this analysis would help policymakers and the health professionals to reduce the cases and fatality rate depending on weather forecast techniques and fight this pandemic using restricted assets.

## 1 Introduction

Coronavirus disease 2019 (COVID-19) is an infectious disease due to coronavirus, a newly observed RNA virus that changed into previously called severe acute respiratory syndrome-coronavirus-2 (SARS-CoV-2) [1, 2, 3]. It causes infection of the respiration tract in human beings and animals, ensuing in fever, cough, and cold. Patients may die from acute respiratory distress syndrome or pneumonia [4]. Coronaviruses are beta coronaviruses that belong to the Orthocoronavirinae subfamily and the Coronaviridae family. Coronavirus receives its call from the Latin phrase corona, which means that crown or wreath. Coronaviruses were first detected within the 1930s in North Dakota in domesticated chickens with an acute respiratory tract infection [3].

HCoV-229E, HCoVOC43, HCoV-HKU1, and HCoV-NL63 are seven coronavirus species recognized to contaminate human beings and cause disease. They are regularly mild and cause typical cold symptoms. Middle East respiratory syndrome-associated coronavirus (MERS-CoV), excessive acute respiratory syndrome coronavirus (SARS-CoV), and severe acute respiratory syndrome coronavirus 2 (SARS-CoV-2) are the other three human coronaviruses that motive probably severe symptoms and have been observed in 2012, 2002, and 2019 [5, 6]. The Chinese Center for Disease and Prevention diagnosed the first instance of COVID-19 from a patient's throat swab on December 8, 2019, in Wuhan, Hubei Province, China [7]. Since the improvement of the COVID-19 disease in China, it has speedily grown into a worldwide hazard, and the World Health Organization has categorized it as a pandemic (WHO).

This disease has unfolded in 228 nations and territories worldwide, with 544, 433, 589 confirmed cases and 6,341,131 deaths (World Health Organization statistics as of June 20, 2022). The mortality rate is high worldwide due to COVID-19 infection. The mortality rate per 1 million people is approximately 813.5 [7,8].

The highest confirmed COVID-19 cases and deaths were reported in the United States of America, with 88, 004, 073 and 1,038,323, respectively. The second highest confirmed cases have been reported in India, with 43, 311, 049 confirmed cases. The third highest deaths were recorded at 525,873. The third highest confirmed cases have been reported in Brazil, with 31, 704, 193 cases, and the second-highest deaths are 669,109 [8]. In the European region, highest number of confirmed cases was found from France (30, 079, 458), Germany (27, 211,866), UK (22, 472, 503), Russia (18, 400, 927), South Korea (18, 280, 090), Italy (17, 896,065), Turkey (15, 085, 742) as well as highest number of death were reported in European region from Russia (380,517), UK (179,537), Italy (167,780), France (149,039), Germany (140,292), Poland (116, 393) and Spain (107,482) [8]. In the African region, the majority of confirmed cases/death were found in South Africa (3,986,601/101,604), and in the Eastern Mediterranean region, the highest cases/death were found in Iran (7,234,988/141,366). India reported highest number of cases/deaths in South-East Asia region. The confirmed number of cases in Bangladesh was 1,957,200, with 29,131 deaths from June 20, 2022 [8].

About 66.3% of the world population has received at least one dose of a COVID-19 vaccine. 11.99 billion doses have been administered globally, and 7.25 million are now administered daily. The highest number of vaccinated covered by China, 3.4 billion and followed by India (1.96 billion), USA (592.27 million), Brazil (449.34 million), Indonesia (417.52 million), Japan (287.42 million), Bangladesh (275.48 million), Pakistan (259.29 million) and so on. Only 17.8% of people in low-income countries have received at least one dose [9].

The association of COVID-19 with meteorological parameters such as humidity, temperature, precipitation, wind speed, air pressure, rainfall, and sunlight has been examined in previous studies [10, 11, 12, 13, 14, 15]. However, a weak association between COVID-19 and weather variables was found in the available studies [16, 17]. A bidirectional relationship between COVID-19 and weather variables was also seen in a previous systematic review [18]. The recent meta-analysis and systematic review result also found a significant association between weather variables and COVID-19 transmission [19, 20]. Several studies reported that maximum temperatures have low effects on COVID-19 transmission [11, 21, 22] while contradicting results were also found [23]. Weather variables, including temperature, must be appreciably undoubtedly positively correlated with the transmissibility of COVID-19 in Singapore, Brazil, Indonesia, Japan, and Norway but significantly negatively correlated in New York City, Iran, Bangladesh, and China [24]. One study revealed that other weather variables like relative humidity (RH) and rainfall were negatively associated with new daily cases and deaths [25, 26, 27]. A positive association was found between COVID -19 cases and relative humidity in a study [28], whereas no correlation between them was also seen in another study [29]. A researcher has also proven a significant relationship between COVID-19 cases and wind speed. The impact of wind speed has a significant positive effect on COVID-19 cases [30, 31]; however, a negative result was also found [32]. Another literature review concluded that sunlight significantly impacts covid 19 cases [27]. The relationship between weather variables and COVID-19 transmission will also be due to season modifications and geographical location [33, 34, 35]. Due to conflicting findings about the association between weather variables and COVID-19 cases and deaths, it is imperative to compile all available data for study in order to identify a consistent effect of weather variables on the COVID-19 cases and deaths. Based on the previous literature, it may be said that the discrete conclusion is yet to be drawn on the prospective role of the weather variables on COVID-19 worldwide. Therefore, it wishes more studies on this subject matter in different world regions, including Bangladesh.

The purpose of this study is to review the correlation between weather variables (such as temperature, rainfall, relative humidity, precipitation, dew-point, air pressure, wind speed, and sunlight) and COVID-19 cases as well as deaths and to review the existing findings in a meta-analysis.

## 2 Methods

This study has shown a Meta-analysis of the impacts of weather variables (temperature, relative humidity, rainfall, wind speed, precipitation, air pressure, dew point, sunlight) on the daily number of confirmed COVID-19 cases COVID-19 death. Thus, this study performed proper inclusion and exclusion based on available literature on the correlation between weather variables and the COVID-19 cases/COVID-19 deaths. After exclusion and inclusion from the selected relevant articles, this study considers the average value of the following weather variables: temperature, relative humidity, rainfall, wind speed, precipitation, air pressure, dew point, and sunlight to find the correlation between death and incidence of the COVID-19. The study performed forest and funnel plots to investigate heterogeneity and publication bias. Lastly, the study conducted a sensitivity analysis to find the most prominent study on the pooled result.

### 2.1 Search strategy

Five electronic databases such as PubMed, Science Direct, Scopus, Ovid (Medline), and Ovid (Embase) were searched to conduct the literature survey from January 01, 2020, to February 03, 2022, using a set combination of keywords to search the desired articles. The detailed search strategy of different databases is described in supplementary files (Appendix A). As for example, the search term for PubMed was “((((((coronavirus [Medical Subject Headings (MeSH Terms)]) OR COVID-19 [MeSH Terms]) OR SARS-CoV-2 [MeSH Terms]) OR Severe Acute Respiratory Syndrome related coronavirus [MeSH Terms])) AND correlation) AND (((weather variable) OR weather variable) OR weather variables)” to find out the potential paper.

### 2.2 Eligibility criteria of the study

This study included the articles assessing the correlation between the average value of the weather variables (temperature, relative humidity, rainfall, wind speed, precipitation, air pressure, dew point, and sunlight) and COVID-19 incidence, as well as the correlation between weather variables and COVID-19 deaths as the primary outcome of interest. Articles that reported a correlation between weather variables and COVID-19 cases/incidence or COVID-19 deaths/fatalities were included in this study. Studies that didn't report any correlation between the weather variables and the COVID-19 cases or COVID-19 deaths were excluded from this study. This analysis considered cross-sectional, time series, or case study designs. Randomized controlled trials, cohort studies, case-control studies, case report study designs, letters to editors, systematic review articles, editorials, and short communication were excluded from this research. This study only considered articles that included daily confirmed COVID-19 cases, deaths, and weather variables. Human-based studies and English language writing articles were included in this study. This study has included only peer-reviewed and published articles and excluded unpublished articles due to data uncertainty.

### 2.3 Data extraction process and study quality assessment

In Rayyan-Intelligent Systematic Review software, all identified possible articles were entered. After entering all the articles in the software, duplicate articles were detected and then removed the duplicate one. After removing the duplicate, two reviewers independently screened the title and the abstract based on the search strategy. Those articles selected for inclusion were finally full-screened by the two independent reviewers. Controversial matters were resolved after discussion. The extracted data included based on weather/weather parameters (such as temperature, relative humidity, rainfall, wind speed, precipitation, air pressure, dew point, sunlight) and the daily number of COVID-19 cases as well as deaths, author name, country, place of data collections, year of publication, time, and study design. PRISMA checklist was used to present the results of this analysis [36]. Using the Joanna Briggs Institute (JBI) tool to assess the quality of the articles [37]. JBI tools contain a total of eight questions, such as; Q1: Where were the criteria for inclusion in the sample clearly defined, Q2: Were the study subjects and the setting described in detail, Q3: Was the exposure measured validly and reliably, Q4: Were objective, standard criteria used for measurement of the condition, Q5: Were confounding factors identified, Q6: Were strategies to deal with confounding factors stated, Q7: Were the outcomes measured validly and reliably And Q8: Was appropriate statistical analysis used. All the questions from JBI were examined against all the articles. In this study, the answer was taken in dichotomous “Yes (1)” and “No (0)”. Overall quality was assessed with 6, and above “yes” responses, then it was considered a high-quality publication, 4 and 5 considered moderate. Less than 4 were deemed low-quality publications from the 8 points [38]. A quality assessment table for all included articles was given in the supplementary file (Table S1).

### 2.4 Statistical analysis

Data analysis was conducted using R programming software and Microsoft Excel. The pooled correlation (r) and 95% confidence interval (CI) were calculated using both fixed effects and random effects models [39, 40]. The pooled correlation was used to calculate the correlation between weather variables and the COVID-19 cases and deaths. Chi-square test statistics (Q), I^2^, and $\tau^{2}$ test was used in this analysis to examine the between-study heterogeneity [41]. To see the heterogeneity among the included studies, this study used forest plots. Existing heterogeneity was identified through subgroup analysis based on the continent. A single study's effect on the overall result was observed by doing sensitivity analysis. By using a funnel plot, publication bias is detected [42].

## 3 Results

### 3.1 Search results and study characteristics

**Figure 1:**
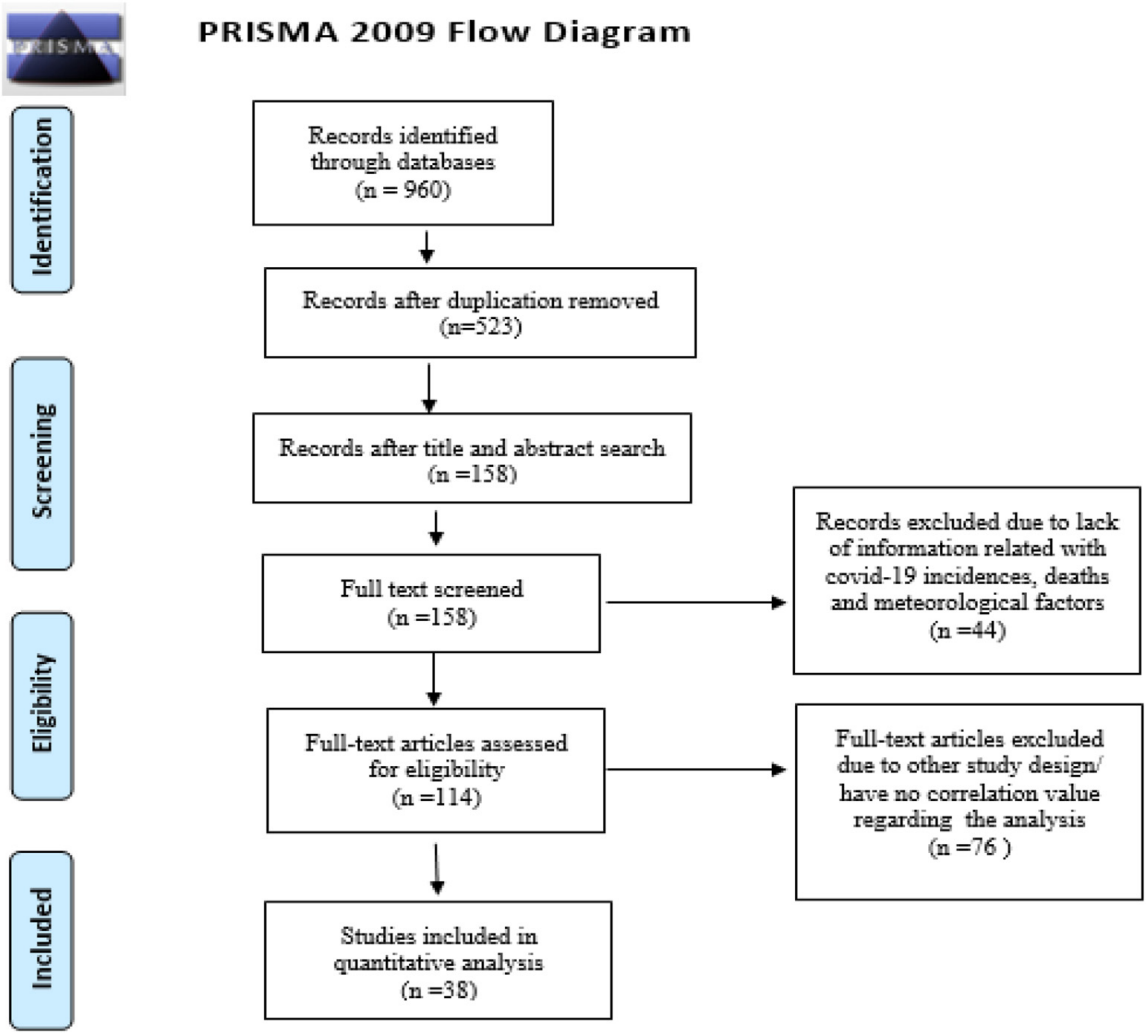
PRISMA flowchart for search strategy and the process of selecting articles.

960 articles were found from five databases Scopus, Science Direct, PubMed, Ovid (Medline), and Ovid (Embase). Of the total articles, 523 articles were identified after duplication was removed, and from those remaining articles, 158 articles were identified through title and abstract. One hundred fourteen articles were selected for full-text assessment, while 76 articles were excluded due to lack of proper information. Finally, 38 publications were included in this meta-analysis (Figure 1). The characteristics of the included studies are detailed in (Table 1). The articles included studies in countries worldwide belonging to Bangladesh, India, Indonesia, China, Jordan, Malaysia, Japan, Spain, Italy, USA, Norway, Poland, Brazil, Saudi Arabia, Singapore, Taiwan, Russian federation, UK, Australia, and Africa. The article mainly used Spearman's rank correlation and Pearson's correlation coefficient values for analysis purposes. A cross-sectional time series or case study was included in this article.

**Table 1:** Characteristics table for included studies.

Author	Year	Country	Study period	Total days	Technique used	Quality Score	Variable	Results/Findings
Asyary A & Veruswati M [43]	2020	Indonesia	2nd March to 10th April 2020	40	Spearman's correlation	6	COVID-19 positive cases, deaths, patients who recovered, and sunlight exposure.	The study's findings showed that sunlight exposure was associated with recovery from COVID-19.
Menebo MM [44]	2020	Oslo, Norway	February 27–May 2, 2020	66	Spearman's correlation	5	Temperature (°C), precipitation level (mm), and wind speed (m/s).	Maximum temperature, average temperature, and precipitation level were significantly correlated with the COVID-19 pandemic.
Dogan B et al. [45]	2020	New Jersey, United States	1st March-7th July 2020	129	Spearman's correlation	8	COVID-19 positive cases, temperature, humidity, air quality.	Temperature (◦C) was found to have a negative correlation, while humidity and air quality positively correlate with daily new cases of COVID-19 in New Jersey.
Zoran MA et al. [46]	2022	Madrid, Spain	15 February 2020–25 July 2021	527	spearman's correlation	8	Average temperature (T), average relative humidity (RH), and average wind speed	A significant negative correlation was found between air temperature, Planetary Boundary Layer height, and surface solar irradiance with daily new COVID-19 incidence and deaths.
Tosepu R et al. [47]	2020	Jakarta, Indonesia	January–March 29, 2020	89	Spearman's correlation	5	Maximum temperature, average temperature, minimum temperature, rainfall, humidity	Average temperature (°C) was significantly associated with the COVID-19.
Bashir MF et al. [48]	2020	New York, USA	March 1, 2020–April 12, 2020	43	Kendall, Spearman correlation	6	Temperature, humidity, wind speed, air quality, and rainfall, COVID-19 cases	Average temperature, minimum temperature, and air quality were significantly associated with the COVID-19 pandemic
Zhang Z et al. [49]	2020	China	January 24 to February 29, 2020	37	Kendall, Spearman correlation	7	Maximum temperature, average temperature, minimum temperature, humidity, wind speed, and COVID-19 cases	Results revealed a kind of nonlinear dose-response relationship between temperature and coronavirus transmission
Rosario DKA et al. [31]	2020	Rio de Janeiro, Brazil	March 12-April 28, 2020	48	Spearman's correlation	6	Temperature, relative humidity, wind speed, and Rainfall	Temperature (maximum and average) and wind speed showed a negative correlation with COVID-19
Ishii T et al. [50]	2021	Sendai City, Japan	July 2020 and April 2021	304	Spearman's correlation	6	Temperature, humidity, wind speed, sunlight	COVID-19 infection significantly correlated with lower atmospheric humidity and higher wind speed.
MEO SA et al. [51]	2020	Russia, United Kingdom, Spain, Italy, Germany, Turkey, France, Belgium, Netherlands and Belarus	Jan 27, 2020 to July 17, 2020	173	Pearson correlation, s, Linear Regression Analysis	6	Temperature, humidity	A significant inverse correlation was observed between temperature and humidity and the number of cases and deaths.
Islam ARMT et al. [52]	2020	Bangladesh	March 8 to May 31, 2020	85	Pearson's correlation	6	Maximum temperature, average temperature, minimum temperature, relative humidity, wind speed, and COVID-19 cases	The results showed that the minimum and mean temperature, wind speed, relative humidity, and absolute humidity significantly correlated with the number of COVID-19 confirmed cases.
Gautam S et al. [53]	2021	India	January 2020 to August 2020	244	Kendall Spearman rank correlation	7	Temperature (Max, min, ave), relative humidity (max, min, ave), COVID-19 cases, and death	Association between air pollution, COVID-19 confirmed cases, and weather variables (T and RH) is plausible.
Shahzad K et al. [54]	2020	Spain	29 February to 17 July 2020	140	Pearson Spearman correlation	7	temperature, COVID-19 cases	Overall empirical results show that the temperature may not be a determinant to induce COVID-19 spread in Spain, while the rising temperature may reduce the virus transmission
Lasisi TT & Eluwole KK [55]	2020	Russian Federation	March 21 and May 28, 2020	69	Spearman's correlation	6	Maximum temperature, minimum temperature, average temperature, rainfall	findings indicated a stronger correlation between average temperature and recorded significant correlations for the other temperature variants.
Sahoo MM [56]	2020	India	30th Jan 2020 to 23rd Apr 2020	85	Spearman's correlation and GAM Model	8	mean temperature, relative humidity, air pressure, rainfall, wind speed, and wind direction	There are significant correlations between air pollutants and weather variables with COVID-19-infected cases
Sarmadi M et al. [57]	2021	UK	March 21 to November 13, 2020	238	Spearman, Pearson	7	temperature, humidity, precipitation, wind speed	The primary results of this study reveal that wind speed, humidity, the temperature may account for geographical variation in the spread of SARS-CoV-2 across the UK
Suhaimi NF et al. [58]	2020	Kuala Lumpur, Malaysia	1 January to 21 April 2020	112	Spearman correlation	5	Relative humidity, ambient temperature, wind speed, and solar radiation	Spearman's correlation test showed that COVID-19 cases were positively correlated with relative humidity.
Abdelhafez E et al. [59]	2021	Jordan	March 15 to August 31, 2020	170	Spearman, sensitivity analysis	7	Average daily temperature, relative humidity, wind speed, pressure	It was found that the most effective weather parameter in the active cases of COVID-19 was the maximum temperature, followed by wind speed and pressure.
Gonçalves J et al. [60]	2021	Ljubljana, Slovenia	4th of March 2020 and 30th of September 2020	211	Spearman rank correlation	6	Minimum, average, and maximum daily temperature, precipitation; relative humidity; sun duration in hours	Daily new COVID -19 cases and weather data did not show any significant association while relative humidity showed the highest correlation coefficient.
MEO SA et al. [61]	2020	Africa	Feb 14, 2020 to August 2, 2020.	140	Pearson correlation	5	Mean temperature, humidity, number of daily COVID-19 cases, daily deaths	An increase in relative humidity and temperature was associated with a decrease in the number of daily cases and deaths due to the COVID-19 pandemic in various African countries
Bolaño-Ortiz TR et al. [62]	2020	Argentina	5 March to 31 May 2020	88	Spearman rank correlation	7	Temperature (max, min, ave), humidity, and accumulated Rainfall	Findings showed a significant correlation between weather and air quality variables and COVID-19 cases.
Zheng Z et al. [63]	2021	China	December 20, 2019, to March 10, 2020.	82	Spearman and linear regression	8	Temperature, wind speed, and absolute humidity.	The correlations between COVID 19 and weather variables were significantly different. High humidity, low wind speed, and relatively lower air temperature.
Abraham J et al. [64]	2021	Victoria, Australia	25 January to 31 October 2020	281	Linear regression, Pearson, Spearman correlation	8	Temperature, humidity, and rainfall	Minimum temperature had a significant negative correlation and a positive effect 21 days later. No significant correlation was found between maximum temperature and rainfall.
Rendana M [32]	2020	Jakarta, Indonesia	March 2, 2020 to May 13, 2020	73	Spearman rank correlation	6	Temperature, humidity, wind speed, wind direction, rainfall and sunlight hours	The study reveals that a low wind speed is significantly correlated with higher COVID-19 cases, similarly to low temperatures and sunlight hours.
Pani SK et al. [65]	2020	Singapore	January 23 to May 31, 2020	130	Spearman and Kendal rank correlation	6	Temperature, relative humidity, dew point (max, min, ave), wind speed (max, min, ave).	Temperature, dew point, relative humidity, absolute humidity, and water vapor showed a significant positive correlation with the COVID-19 pandemic
Zoran MA et al. [66]	2020	Milan, Italy	1 January–30 April 2020	121	Pearson correlation	7	Temperature, humidity, COVID-19 cases, deaths	COVID-19 cases outbreak in Milan is positively associated with average surface air temperature and inversely related to air relative humidity.
Alkhowailed M et al. [67]	2020	Saudi Arabia	May 1, 2020–Jun 7, 2020	38	Spearman rank correlation	6	Temperature, humidity, air pressure, wind spread	The number of COVID-19 positive cases increases due to the decrease in temperature or humidity, whereas decreased wind speed was also associated with positive cases.
Ismail IMI et al. [68]	2022	Saudi Arabia	1st March to August 31, 2020	184	Spearman rank correlation	8	Daily temperature, relative humidity, and dew point	Daily COVID 19 infections showed a positive relationship with temperature between 23 and 34.5 °C and relative humidity ranging from 30 to 60%; a negative relationship was found below and above.
Kumar G & Kumar RR [69]	2020	Mumbai, India	April 27 till July 25, 2020	90	Spearman rank correlation	6	Temperature, humidity, dew point, wind speed	A significant correlation between COVID-19 was found with temperature, dew point, Relative humidity.
Wang Q et al. [70]	2021	China	January to October 2020	305	Spearman, linear regression	7	Temperature, wind speed, precipitation, air pressure	The daily average temperature, wind speed, precipitation, and new COVID-19 cases were negatively correlated
Sangkham S et al. [71]	2021	Bangkok, Thailand	January 1 to March 30, 2020	90	Kendal and Spearman's Rank correlation	7	Temperature, wind speed, rainfall, humidity	Weather parameters such as temperature, relative humidity, and wind speed are positively associated with daily confirmed COVID-19 cases in the BMR.
Werner PA et al. [72]	2021	Poland	March 2020 till July 2021	519	Pearson's correlation	6	Temperature	The hypothesis under consideration concerns an increase in the number of COVID-19 cases as temperature decreases
Sharif N et al. [73]	2021	Japan	January 2020 to February 2021	425	Spearman's rank correlation	6	Temperature (max, min, ave), precipitation, wind velocity, rainfall, and Relative humidity	Relative humidity had the highest correlation with the case fatality rate.
Sharif N and Dey SK [74]	2020	Bangladesh	07 March 2020 to 14 August 2020	161	Spearman rank correlation	6	Temperature (max, min, ave), wind speed, rainfall, relative humidity	Among metrological parameters, the average temperature strongly correlated with the cases.
Mofijur M et al. [75]	2020	Bangladesh	1 Ma y 2020 to 31 Ma y 2020	31	Spearman rank correlation	8	Temperature (max, min, ave), wind speed, rainfall, relative humidity	Minimum temperature and average The temperature had a significant relationship with new COVID-19 cases.
Chang SA et al. [76]	2021	Taiwan	May 1–May 28, 2021	28	Spearman rank correlation	6	Maximum temperature, wind speed, relative humidity	Maximum daily temperature significantly Positively correlated with daily new COVID-19 cases.
Bilal et al. [77]	2021	USA	March 2, 2020, to September 17, 2020,	200	Spearman, Kendal rank correlation	7	Maximum temperature, rainfall, relative humidity, daily new cases, daily new deaths of COVID-19	Temperature, humidity, environmental quality index, and rainfall are significant Variables related to the COVID-19 pandemic in the USA's top 10 most affected states.
Gautam S et al. [78]	2021	India	January 2020 to August 2020	243	Spearman, Kendal rank correlation	8	Temperature (max, min, ave), relative humidity (max, min, ave), daily new cases, daily new deaths of COVID-19	Temperature and relative humidity are significantly correlated with COVID-19 cases and deaths.

### 3.2 Overall outcomes

**Figure 2:**
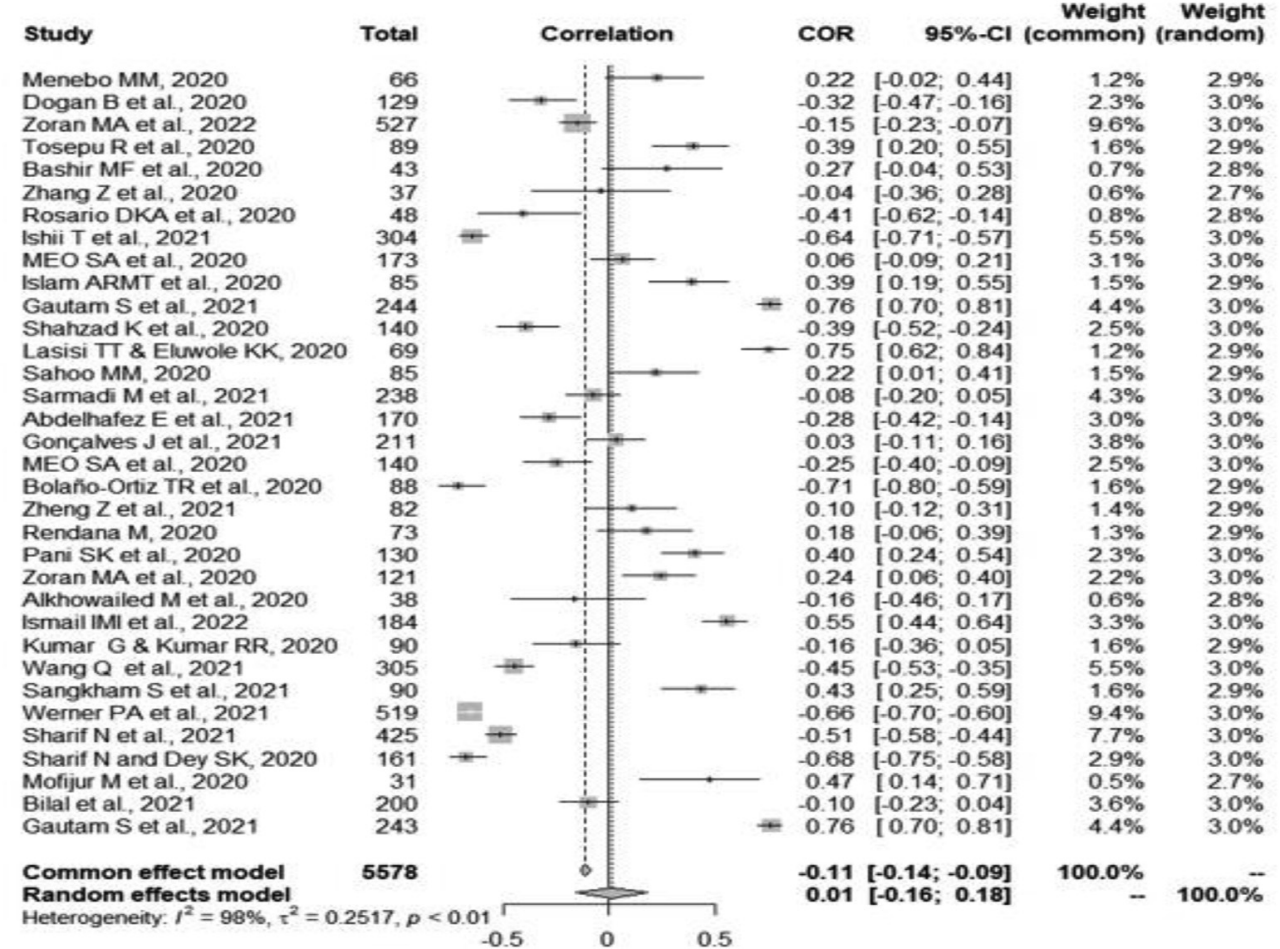
Forest plot of COVID-19 incidence and temperature.

The overall outcomes of this study have shown in Table 2. This study showed a negative correlation between temperature and the COVID-19 incidence for the fixed-effect model -0.113∗∗∗ (95% CI: -0.139, -0.087), but it was insignificant for random effect model 0.009 (95% CI: -0.161, 0.179) from the 34 studies (Figure 2). In the case of temperature and death, the correlation was also negatively correlated for the fixed-effect model -0.094∗∗∗ (95% CI: -0.128–0.060) from the 15 studies (given in supplementary file) (Fig. S1). We have found 30 studies about the correlation between relative humidity and the COVID-19 incidence. There exists a significant negative correlation between relative humidity and the incidence of COVID-19 for the fixed-effect model -0.019∗∗∗ (95% CI: -0.048, 0.011), but for the random effect model, the correlation was insignificant at 0.015 (95% CI: -0.115, 0.144) (Fig. S2). On the other hand, the correlation between relative humidity and death was significant for the fixed-effect model 0.059∗∗∗ (95% CI: 0.022, 0.095) and insignificant for the random effect model -0.059 (95% CI: -0.301, 0.191) (Fig. S3). This study has shown that wind speed and COVID-19 incidence had a significant positive correlation for the fixed-effect model 0.071∗∗∗ (95% CI: 0.038, 0.103) but insignificant for the random effect model (Fig. S4). For the fixed-effect model, death was significantly negatively correlated with the wind speed -0.048∗∗ (95% CI: -0.096, 0.001) (Fig. S5). One weather variable, rainfall, is insignificant with COVID-19 incidence for both fixed-effect and random effect models (Fig. S6). Still, it was significant with COVID death for fixed-effect model -0.158∗∗∗ (95% CI: -0.224, -0.090) (Fig. S7). But precipitation is significantly negatively correlated with COVID-19 cases for the fixed-effect model -0.143∗∗∗ (95% CI: -0.209, -0.075) and insignificant for the random effect model (Fig. S8). In both the fixed and random effect models, air pressure is significantly negatively correlated with COVID-19 cases (Fig. S9). Dew point was reported a positive association with the COVID-19 cases for fixed-effect model 0.115∗∗∗ (95% CI: 0.039, 0.189) (Fig. S10), but death was insignificant for both models (Fig. S11). Sunlight also negatively correlated with the COVID-19 incidence as well as death for fixed-effect model -0.277∗∗∗ (95% CI: -0.332, -0.220) (Fig. S12), and -0.271∗∗∗ (95% CI: -0.348, -0.190) (Fig. S13). Heterogeneity (I^2^) was mostly observed COVID-19 incidence with the temperature, sunlight, relative humidity, dew point, and wind speed variables, i.e., 97.9%, 97.5%, 96%, 93.7%, and 91.3%, respectively, and death with temperature, and relative humidity 99.1%, and 98.5% respectively.

**Table 2:** Correlation between Weather variables and COVID-19 incidence and deaths.

COVID-19	Variables	No of Studies	Total no of days	Pooled Correlation [95% CI]		Test for heterogeneity		
				FEM	REM	Q statistic	$\tau^{2}$	$I^{2}$
**Incidence**	Temperature	34	5578	-0.113∗∗∗ [-0.139, -0.087]	0.009 [-0.161, 0.179]	1595.23∗∗∗	0.252	97.9%
	Relative Humidity	30	4294	-0.019∗∗∗ [-0.048, 0.011]	0.015 [-0.115, 0.144]	730.00∗∗∗	0.120	96.0%
	Wind Speed	24	3377	0.076∗∗∗ [ 0.042, 0.110]	0.039∗∗∗ [-0.095, 0.172]	275.69∗∗∗	0.099	91.7%
	Rainfall	12	1519	-0.041 [-0.091, 0.010]	-0.052 [-0.144, 0.042]	25.73∗∗∗	0.015	57.2%
	Precipitation	4	823	-0.143∗∗∗ [-0.209, -0.075]	-0.106 [-0.249, 0.042]	11.56∗∗∗	0.017	74.0%
	Air Pressure	5	616	-0.073∗ [-0.152, 0.006]	-0.073∗ [-0.152, 0.006]	3.71	00	0.0%
	Dew Point	5	680	0.115∗∗∗ [ 0.039, 0.189]	0.183 [-0.106, 0.444]	63.54∗∗∗	0.099	93.7%
	Sunlight	5	1053	-0.277∗∗∗ [-0.332, -0.220]	-0.166 [-0.511, 0.225]	159.26∗∗∗	0.195	97.5%
**Death**	Temperature	15	3379	-0.094∗∗∗ [-0.128–0.060]	0.042 [-0.288, 0.362]	1474.38∗∗∗	0.439	99.1%
	Relative Humidity	14	2860	0.059∗∗∗ [0.022, 0.095]	-0.059 [-0.301, 0.191]	891.98∗∗∗	0.224	98.5%
	Wind Speed	8	1676	-0.048∗∗ [-0.096, 0.001]	-0.036 [-0.197, 0.127]	75.21∗∗∗	0.048	90.7%
	Rainfall	5	829	-0.158∗∗∗ [-0.224, -0.090]	-0.107 [-0.268, 0.059]	19.71∗∗∗	0.026	79.7%
	Dew Point	2	422	-0.071 [-0.166, 0.025]	-0.061 [-0.258, 0.141]	4.40∗∗	0.017	77.3%
	Sunlight	3	538	-0.271∗∗∗ [-0.348, -0.190]	-0.137 [-0.474, 0.235]	12.91∗∗∗	0.097	84.5%

∗∗∗ for P value < 0.001, ∗∗ for 0.001 < p value < 0.01 and ∗ for p value < 0.05.

### 3.3 Subgroup analysis

The correlation between weather variables and COVID-19 incidence and death regarding the continent was presented in the subgroup analysis (Table 3). 34 studies were found about the correlation between COVID-19 incidence and temperature on a different continents. The highest negative correlation was found in South America -0.729∗∗∗ (95% CI: -0.901, -0.557) and lowest in North America -0.140∗∗∗ (95% CI: -0.243, -0.037). Positive correlation was found for COVID-19 incidence and temperature in Asia 0.016∗∗∗ (95% CI: -0.022, 0.055) (Fig. S14). The overall correlation between relative humidity and COVID-19 incidence was significant. Heterogeneity of this correlation was found continent-wise. In Asia, the correlation was significant and negatively correlated -0.056∗∗∗ (95% CI: -0.096, -0.016) (Fig. S15). Similarly, the overall correlation between wind speed and COVID-19 incidence was significantly positively correlated. Heterogeneity of this correlation was found continent-wise. The correlation was significantly positive in Asia, but in South America, it was significantly negatively correlated (Fig. S16). Heterogeneity was found among different continents for rainfall and COVID-19 incidence (Fig. S17). Overall precipitation was negatively associated with COVID-19 incidence, and a higher negative correlation was found in Asia (Fig. S18). Widespread precipitation was negatively correlated with COVID-19 incidence, but heterogeneity among the continent was insignificant (Fig. S19). Dew point was positively correlated with COVID-19 incidence; continent-wise, in Europe, it was found to be negative, but in Asia, it was found to be a positive correlation (Fig. S20). The highest negative correlation between COVID-19 cases and sunlight was found in Europe -0.696∗∗∗ (95% CI: -0.832, -0.560) (Fig. S21).

**Table 3:** Subgroup analysis result concerning Continent.

COVID-19	Variables	Continent	No of Studies	Pooled Correlation (95% CI)	Test for heterogeneity		Test for subgroup differences
					Q statistics	I^2^	Q statistics
Incidence	Temperature	Europe	9	-0.214 ∗∗∗ (-0.257, -0.170)	334.30∗∗∗	97.6%	122.39∗∗∗
		North America	3	-0.140∗∗∗ (-0.243, -0.037)	12.05∗∗∗	83.4%	
		Asia	18	0.016∗∗∗ (-0.022, 0.055)	1120.27∗∗∗	98.5%	
		South America	2	-0.729∗∗∗ (-0.901, -0.557)	6.13∗∗	83.7%	
		Africa	2	-0.275∗∗∗ (-0.387, -0.163)	0.09	0.0%	
		Overall	34	-0.113∗∗∗ (-0.139, -0.087)	1595.23∗∗∗	97.9%	
	Relative Humidity	Europe	5	0.029 (-0.026, 0.085)	30.47∗∗∗	86.9%	8.85∗∗∗
		North America	3	0.049 (-0.053, 0.152)	10.43∗∗∗	80.8%	
		Asia	18	-0.056 ∗∗∗ (-0.096, -0.016)	671.02∗∗∗	97.5%	
		South America	2	0.061 (-0.111, 0.233)	3.04∗	67.1%	
		Africa	2	-0.037 (-0.149, 0.075)	6.18∗∗	83.8%	
		Overall	30	-0.019∗∗∗ (-0.048, 0.011)	730.00∗∗∗	96%	
	Wind Speed	Europe	4	0.043 (-0.021, 0.106)	5.49	45.3%	26.44∗∗∗
		North America	1	0.174 (-0.136, 0.484)	0	0%	
		Asia	16	0.119∗∗∗ (0.076, 0.163)	242.29∗∗∗	93.8%	
		South America	2	-0.326 ∗∗∗ (-0.498,-0.154)	1.47	31.8%	
		Africa	1	0.041 (-0.111, 0.193)	0	0%	
		Overall	24	0.077∗∗∗ (0.042, 0.111)	275.69∗∗∗	91.7%	
	Rainfall	Europe	1	-0.293∗∗ (-0.540, -0.046)	0	0%	9.01∗
		North America	2	-0.066 (-0.193, 0.062)	2.54	60.6%	
		Asia	6	0.002 (-0.068, 0.073)	6.96	28.1%	
		South America	2	-0.196 ∗∗ (-0.368, -0.024)	7.22∗∗∗	86.2%	
		Oceania	1	-0.010 (-0.128, 0.108)	0	0%	
		Overall	12	-0.041 (-0.092, 0.010)	25.73∗∗∗	57.2%	
	Precipitation	Europe	3	-0.065 (-0.152, 0.022)	3.02	33.8%	8.54∗∗∗
		Asia	1	-0.277∗∗∗ (-0.390, -0.164)	0	0%	
		Overall	4	-0.144∗∗∗ (-0.212, -0.075)	11.56∗∗∗	74.0%	
	Air Pressure	Europe	1	-0.105 (-0.233, 0.022)	0	0%	0.84
		Asia	3	-0.085 (-0.224, 0.054)	2.87	30.4%	
		Africa	1	-0.015 (-0.167, 0.137)	0	0%	
		Overall	5	-0.074 ∗ (-0.153, 0.006)	3.71	0%	
	Dew Point	Europe	1	-0.173 ∗∗∗ (-0.301, -0.045)	0	0%	30.13∗∗∗
		Asia	4	0.273 ∗∗∗ (0.178, 0.367)	33.40 ∗∗∗	91.0%	
		Overall	5	0.115∗∗∗ (0.039, 0.191)	63.54∗∗∗	93.7%	
	Sunlight	Europe	1	-0.696 ∗∗∗ (-0.832, -0.560)	0	0%	44.08∗∗∗
		Asia	4	-0.181∗∗∗ (-0.250, -0.113)	115.18∗∗∗	97.4%	
		Overall	5	-0.285∗∗∗ (-0.345, -0.224)	159.26∗∗∗	97.5%	
Death	Temperature	Europe	5	-0.428∗∗∗ (-0.477, -0.378)	421.49∗∗∗	99.1%	450.75∗∗∗
		North America	2	0.403∗∗∗ (0.275, 0.530)	0.01	0%	
		Asia	6	0.268∗∗∗ (0.214, 0.323)	602.12∗∗∗	99.2%	
		Africa	1	-0.182∗∗ (-0.349, -0.015)	0	0%	
		South America	1	-0.820∗∗∗ (-1.032, -0.607)	0	0%	
		Overall	15	-0.094∗∗∗ (-0.128, -0.061)	1474.38∗∗∗	99.1%	
	Relative Humidity	Europe	4	0.313∗∗∗ (0.252, 0.373)	302.85∗∗∗	99.0%	109.71∗∗∗
		North America	2	-0.058 (-0.186, 0.069)	1.08	7.1%	
		Asia	6	-0.084∗∗∗ (-0.137, -0.030)	478.34∗∗∗	99.0%	
		Africa	1	-0.216∗∗∗ (-0.384, -0.049)	0	0%	
		South America	1	-0.093 (-0.306, 0.119)	0	0%	
		Overall	14	0.059∗∗∗ (0.022, 0.095)	891.98∗∗∗	98.5%	
	Wind Speed	Europe	3	-0.139∗∗∗ (-0.205, -0.073)	29.56∗∗∗	93.2%	28.78∗∗∗
		North America	1	0.049 (-0.261, 0.359)	0	0%	
		South America	1	-0.316∗∗∗ (-0.529, -0.104)	0	0%	
		Asia	3	0.104∗∗∗ (0.027, 0.181)	16.87∗∗∗	88.1%	
		Overall	8	-0.048∗∗ (-0.096, 0.000)	75.21∗∗∗	90.7%	
	Rainfall	North America	2	-0.011 (-0.138, 0.116)	0.99	0%	7.41∗∗
		South America	1	-0.248∗∗ (-0.461, -0.035)	0	0%	
		Asia	2	-0.215∗∗∗ (-0.303, -0.127)	11.32∗∗∗	91.2%	
		Overall	5	-0.159∗∗∗ (-0.228, -0.090)	19.71∗∗∗	79.7%	

The correlation between weather variables (relative humidity, wind speed temperature, and rainfall) and deaths was significant. Regarding the correlation between temperature and death, South America was highly negatively correlated -0.820∗∗∗ (95% CI: -1.032, -0.607), but on the other hand, North America was highly positively correlated at 0.403∗∗∗ (95% CI: 0.275, 0.530) (Fig. S22). Interesting findings were also found for the variable relative humidity. The correlation was significantly negatively correlated in Asia and Africa, but in Europe, it was significantly positively correlated (Fig. S23). South America found the highest negative correlation between wind speed and death -0.316∗∗∗ (95% CI: -0.529, -0.104) (Fig. S24). For rainfall variables, South America and Asia were significantly negatively correlated with death (Fig. S25).

### 3.4 Publication bias and sensitivity analysis

A funnel plot was used in this analysis to detect publication bias. All variables showed publication bias evidence except COVID-19 incidence and air pressure, COVID-19 death, and dew point (Fig. S26–S39). Sensitivity analysis was used to find the most prominent study on the overall estimates. According to sensitivity analysis, there was no dependence on any one study for the overall estimates of COVID-19 cases and deaths (Fig. S40–S53).

## 4 Discussion

COVID-19 cases and deaths are influenced by social activities that are frequently temperature sensitive. On cold and hot days, people prefer to stay at home, whereas on pleasant days, they prefer to go outside. Temperature variations may indirectly impact COVID-19 incidence and death because the virus spreads more easily in confined spaces. Because viral aerosol dispersal is likely influenced by humidity and temperature, humidity is an essential meteorological factor in COVID-19 transmission. Lowen et al. (2007) conducted 20 experiments at different relative humidity levels ranging from 20% to 80% and temperatures (5 °C, 20 °C, or 30 °C), indicating that both cold and dry conditions favor transmission [79]. Temperature and relative humidity, wind speed, and sunlight are the most crucial weather variables that are strong enough to impact the death and incidence of COVID-19. Correlation parameters were applied to disseminate a clear connection between the weather and COVID-19 in each study that was included. Aside from the heterogeneity and dispersion of actual size effects, this study has an advantage over the fixed and random effect models. Significant forest plots were obtained for the temperature versus the incidence of COVID-19, temperature versus death, relative humidity versus the incidence of COVID-19, relative humidity versus death, precipitation versus the incidence of COVID-19, precipitation versus death, air pressure versus the incidence, dew point versus the incidence, dew point versus death, sunlight versus the incidence, and sunlight versus the death. To ascertain the cause of variance in COVID-19 cases and deaths due to geography, subgroup analysis was done with regards to the continent.

The highest negative correlation between temperature and incidence was found in South America, followed by Africa, Europe, and North America. A significant negative correlation was also found in several studies [31, 46, 51], but a positive correlation between temperature and COVID-19 incidence was found in Bangladesh [52]. The correlation between incidence and relative humidity has been significantly negative in Asia. Italy, Africa, and Saudi Arabia have also found a negative correlation between COVID-19 incidence and relative humidity [61, 66, 67], but a positive correlation was found in Malaysia, Singapore, Thailand, and Bangladesh [52, 58, 65, 71]. An exciting finding was also seen for the correlation between wind speed and incidence, with a positive correlation in Asia and the negative correlation in South America. A negative correlation was also found in Brazil and China [31, 63, 70], and a positive correlation was found in Japan, Bangladesh, and Thailand [50, 52, 71]. In Europe and South America, a negative correlation was found between rainfall and incidence. Only in Asia precipitation was negatively correlated with the incidence. A researcher also found a negative correlation between incidence and precipitation in China [70]. In correlation between dew point and incidence, a significant positive correlation was found in Asia and negative in Europe. The highest negative correlation between sunlight and incidence was found in Europe, followed by Asia. Sunlight was also negatively correlated with Indonesia's incidence [32, 43]. A positive correlation between death and temperature in Asia and North America was found, but a negative correlation was found in Africa and Europe [51, 61]. In this study, the correlation between relative humidity and death was negative in Asia and Africa, but in Europe, it was positive. But MEO SA et al. found a negative correlation between relative humidity and death in Europe [51]. The correlation between Wind speed and death was positive in Asia, but a negative correlation was found in South America and Europe. The correlation between rainfall and death was negative in South America and Asia. An exciting finding was noticed in this study, pooled correlation of incidence and death to the weather variables. Wind speed and incidence were negatively correlated, but wind speed and death were positively correlated. This means the incidence increases, and the death decreases with the increase of wind. In Asia, the death increases but the incidence decrease with the temperature increases. The incidence and death decrease with the increase of relative humidity and incidence and death increase with the increase in wind speed in Asia. In Europe, temperature increases, and the death and incidence decrease. In North America, temperature increases the death increase but incidence decreases. In South America, the incidence and death decrease with increasing temperature, wind speed, and rainfall. In Africa, the incidence and death decrease with the increase in temperature.

A general conclusion is yet to be drawn regarding the correlation between weather variables and COVID-19 incidence and death. Different results have been shown in different continents for a single variable. In order to stop the COVID-19 epidemic, this study suggested that weather experts and medical professionals look more attentively at the data of this paper. To more effectively bolster the findings of our meta-analysis, more study should be done.

### 4.1 Strengths and limitations

There are few publications available summarizing weather and COVID-19 research. This study aimed to investigate the relationship between COVID-19 incidence and mortality and weather variables. The study's strength in including data from several global studies and information from other parts of the world helps us better understand global geographical distribution of transmission and how they interact with weather factors in varied climates. This analysis has also some limitations. English language articles were only included in this study. There are several limitations on weather correlation with COVID-19 cases and death due to the unavailability of much research. Biasness and high heterogeneity were also found in the included studies. Some weather variables could not be included in the analysis due to insufficient of data. Publication bias was also observed. However, this study suggests more research on the correlation between weather and COVID-19 cases and death.

## 5 Conclusion

The relation between COVID-19 incidences and meteorological conditions is complex. The global reach of a pandemic and other factors involved in the COVID-19 pandemic, such as healthcare interventions, public health measures, human behavioral patterns, and socioeconomic factors, make it difficult to examine relationships and correlations with weather variables and COVID-19 incidence dynamics. The majority of studies have found strong correlations between COVID-19 cases and meteorological factors, particularly temperature and humidity, demonstrating the influence of the weather and environment on transmission dynamics. Other factors, including as societal behavior and public health activities, may have a significant impact on future outbreaks, despite the fact that changes in seasonal patterns and weather may increase the incidence and mortality of COVID-19 [79]. This study has found a significant correlation between weather variables and COVID-19 cases and death. Those results would allow the health care specialists or the government's policymakers to make earlier choices before the projected rise in COVID-19 cases based on the weather forecasting technique.

## Declarations

### Author contribution statement

Md. Momin Islam, MS; Farha Musharrat Noor: Conceived and designed the experiments; Performed the experiments; Analyzed and interpreted the data; Contributed reagents, materials, analysis tools or data; Wrote the paper.

### Funding statement

This research did not receive any specific grant from funding agencies in the public, commercial, or not-for-profit sectors.

### Data availability statement

Data will be made available on request.

### Declaration of interests statement

The authors declare no conflict of interest.

### Additional information

Supplementary content related to this article has been published online at https://doi.org/10.1016/j.heliyon.2022.e10333.

## Appendix A Supplementary data

suplimentary file.docx suplimentary file.docx
