# Supplementary material for: Correlation between COVID-19 and weather variables: A meta-analysis
Source: Heliyon. 2022 Aug 18;8(8):e10333. doi: 10.1016/j.heliyon.2022.e10333 (PMC9387066; doi:10.1016/j.heliyon.2022.e10333)
Supplement: suplimentary file.docx [file mmc1.docx]

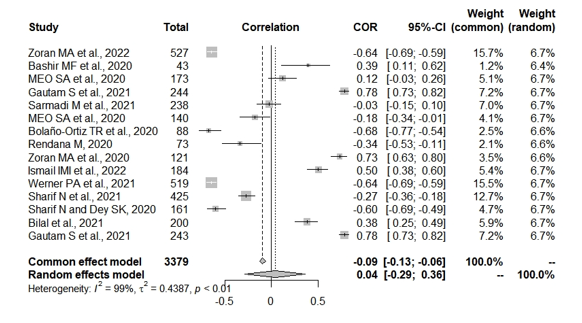


**Fig. S1**: Forest plot of COVID-19 deaths and temperature.


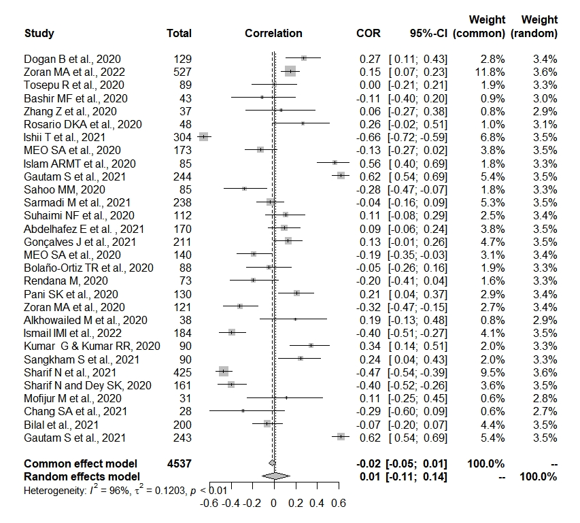


**Fig. S2:** Forest plot of COVID-19 incidence and relative humidity.


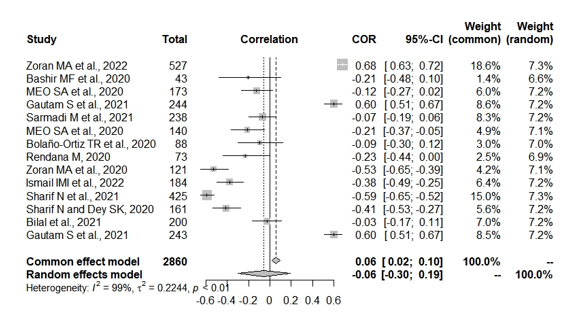


**Fig. S3:** Forest plot of COVID-19 deaths and relative humidity.


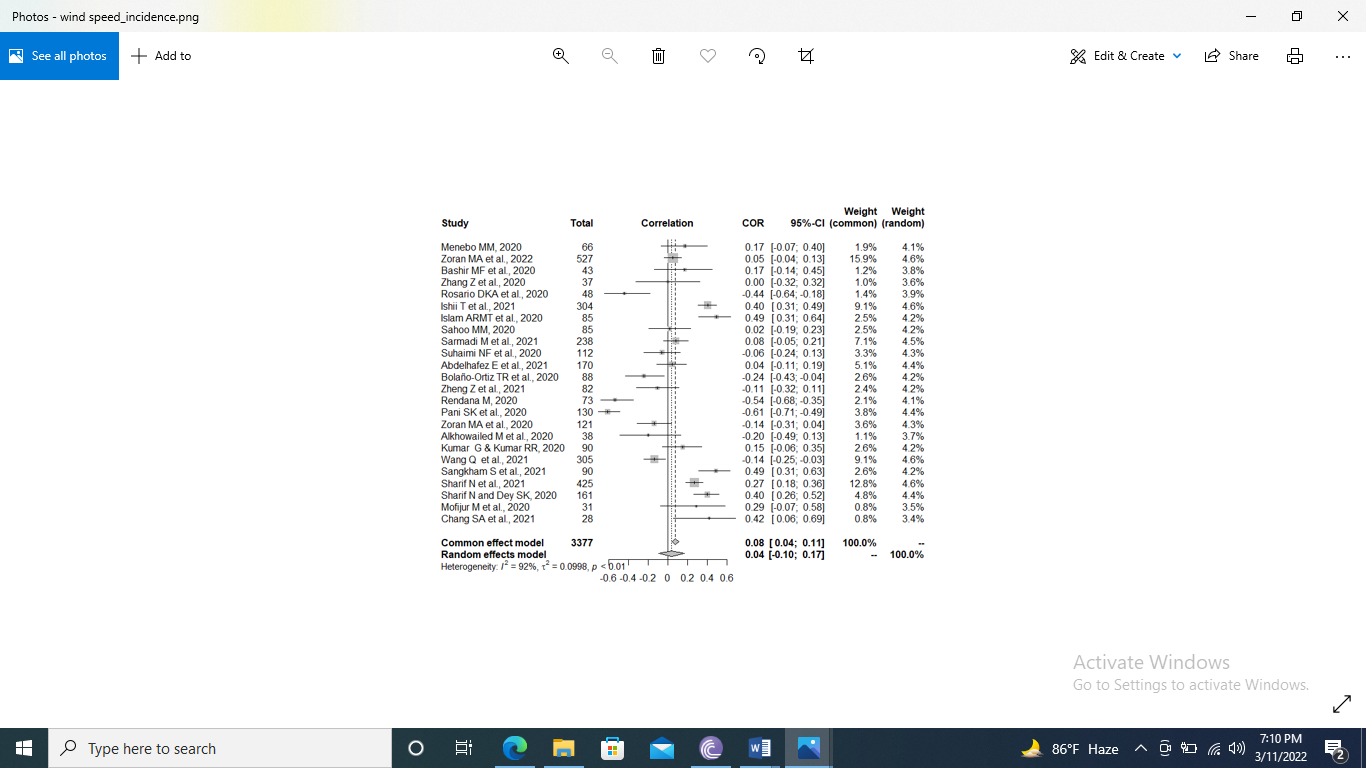


**Fig. S4**: Forest plot of COVID-19 incidence and wind speed.


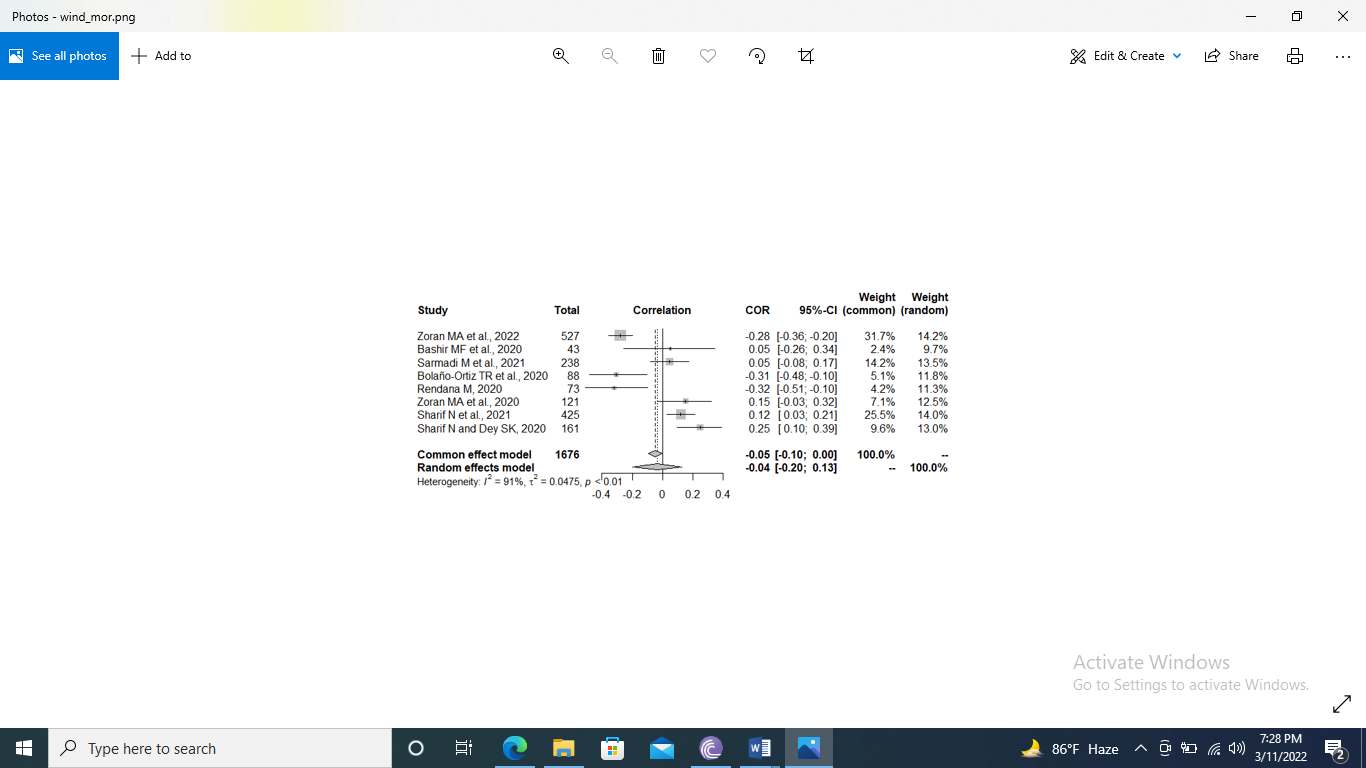


**Fig. S5**: Forest plot of COVID-19 deaths and wind speed.


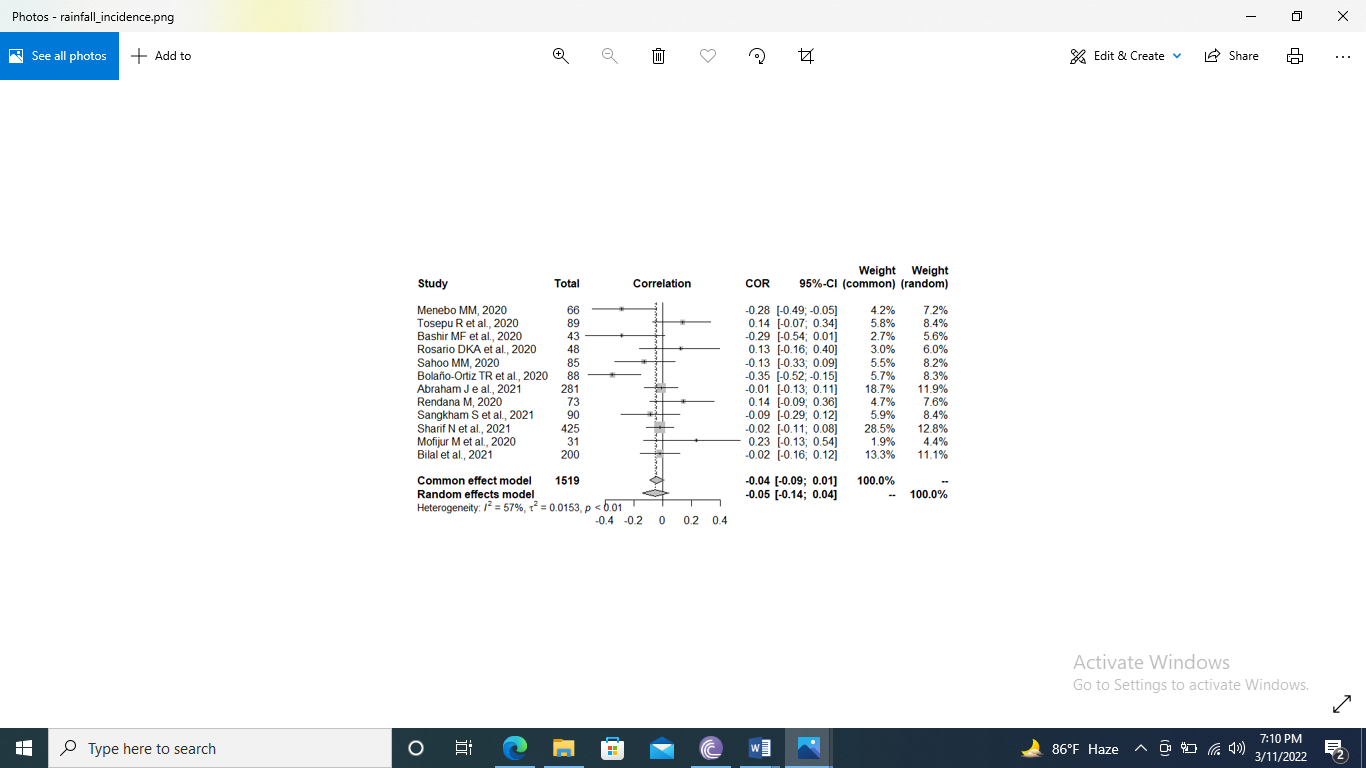


**Fig. S6**: Forest plot of COVID-19 incidence and rainfall.


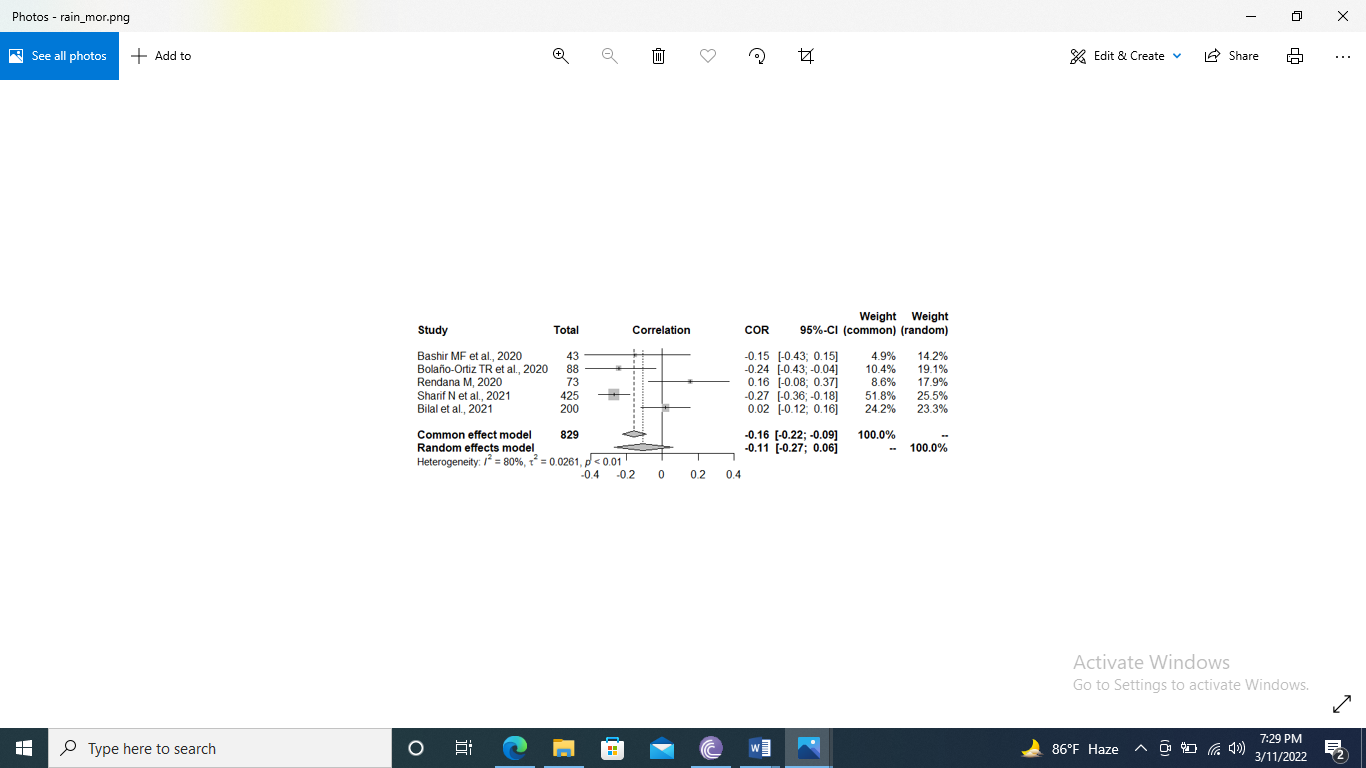


**Fig. S7**: Forest plot of COVID-19 deaths and rainfall.


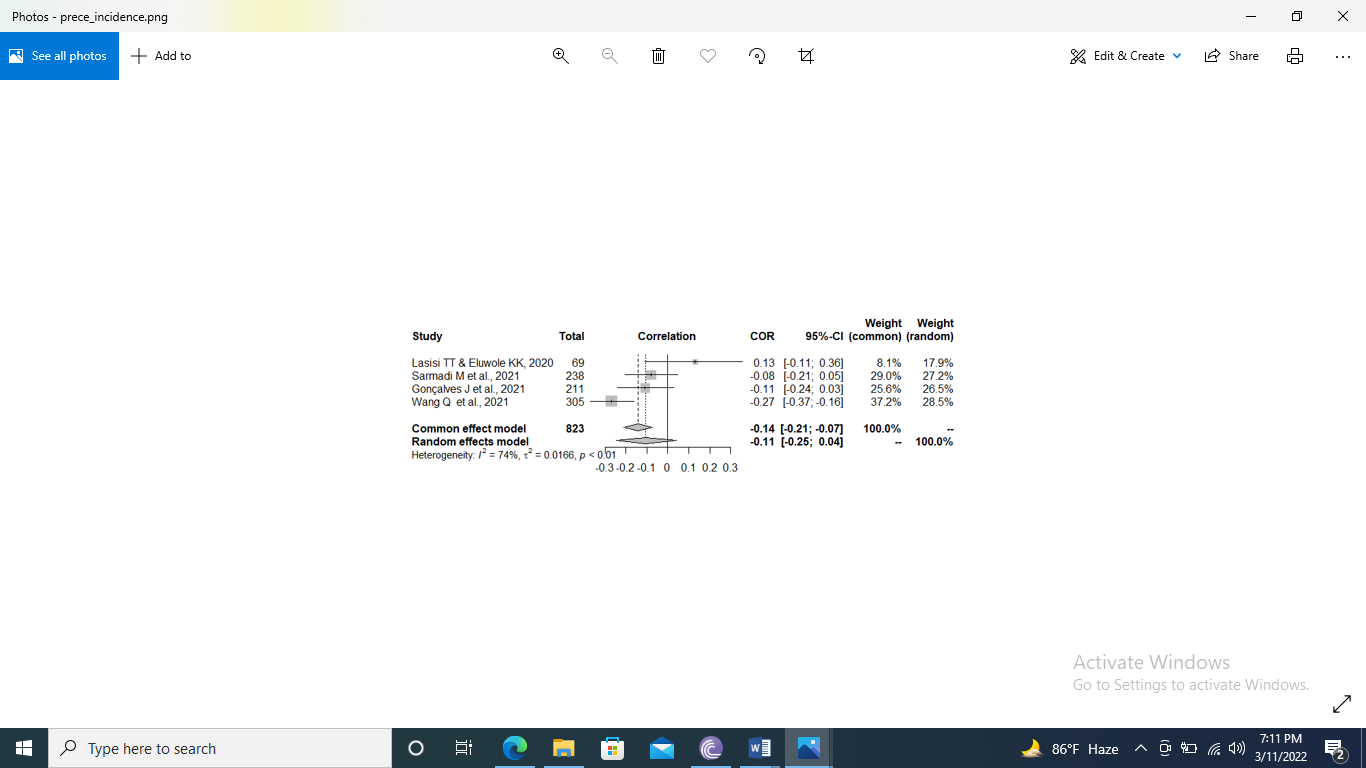


**Fig. S8**: Forest plot of COVID-19 incidence and precipitation.


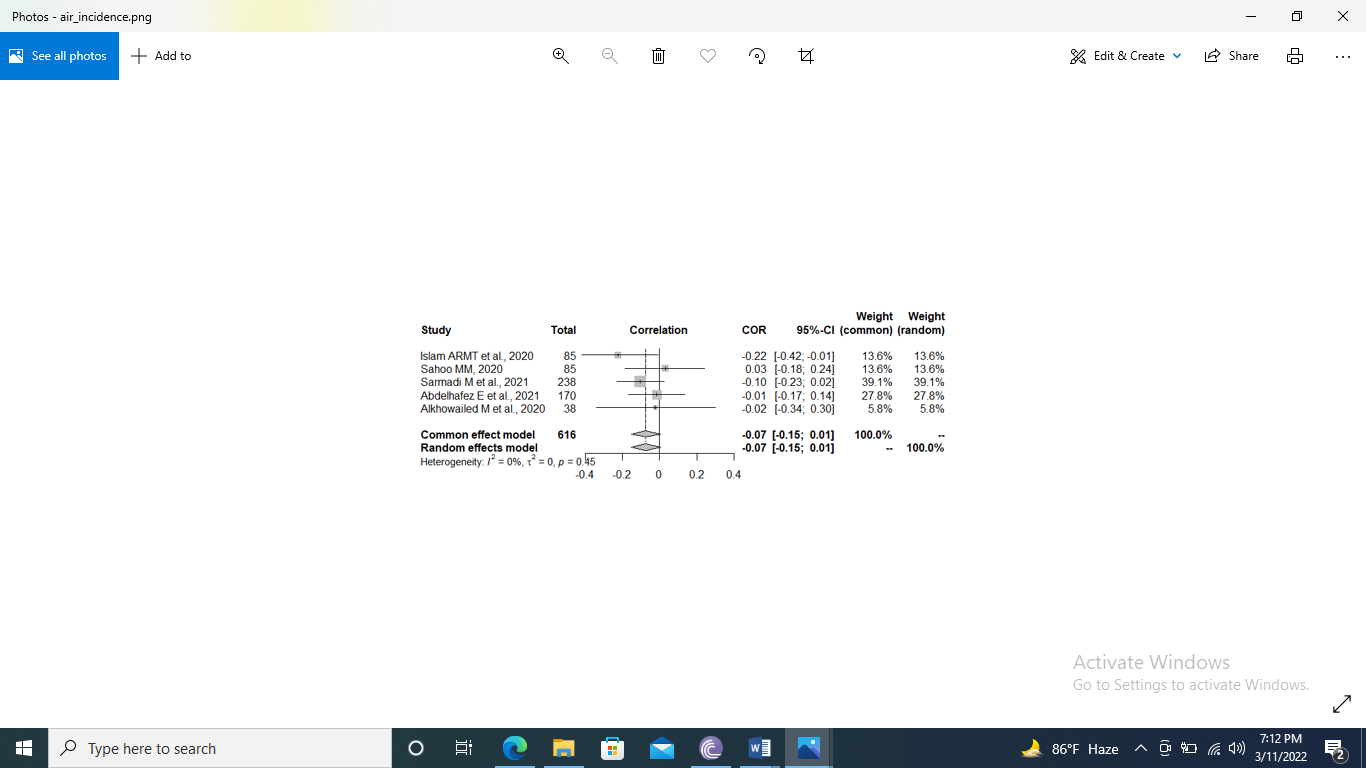


**Fig. S9**: Forest plot of COVID-19 incidence and air pressure.


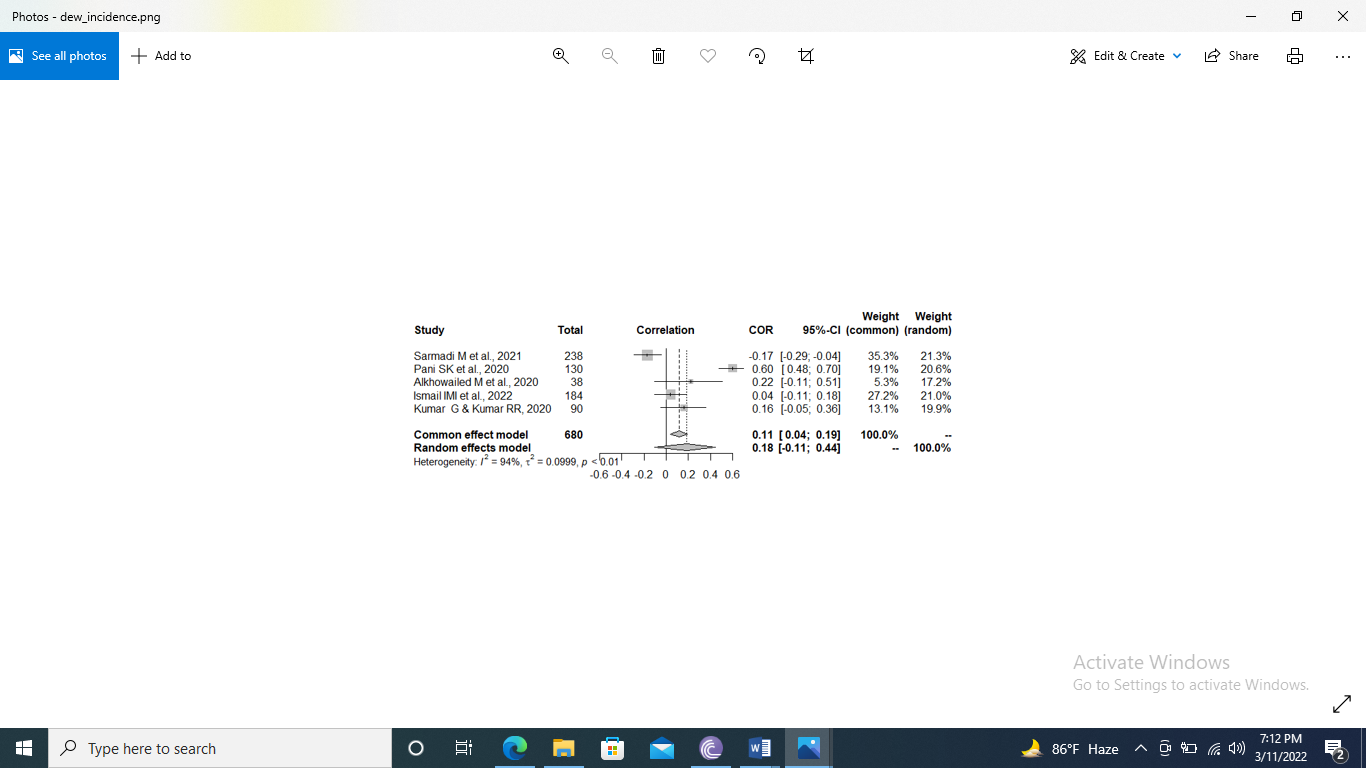


**Fig. S10**: Forest plot of COVID-19 incidence and dew point.


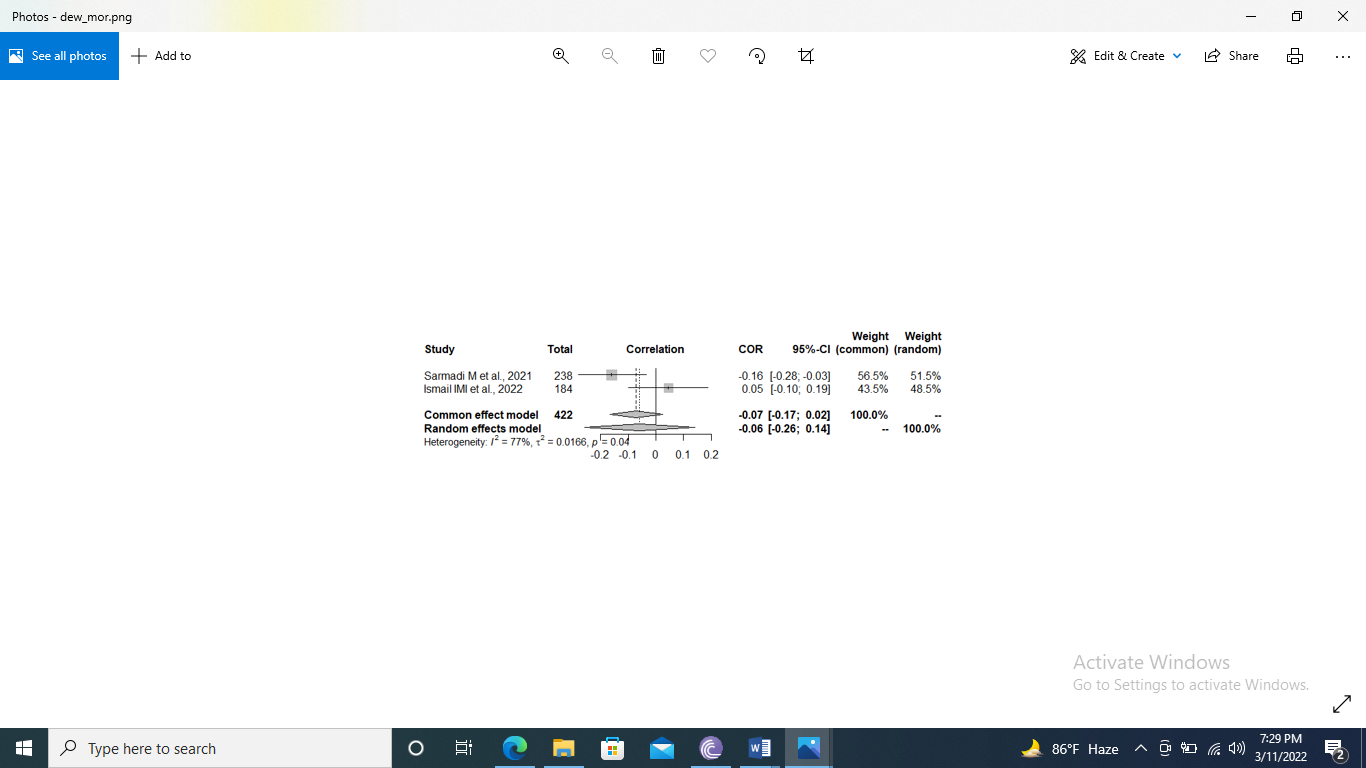


**Fig. S11**: Forest plot of COVID-19 deaths and dew point.


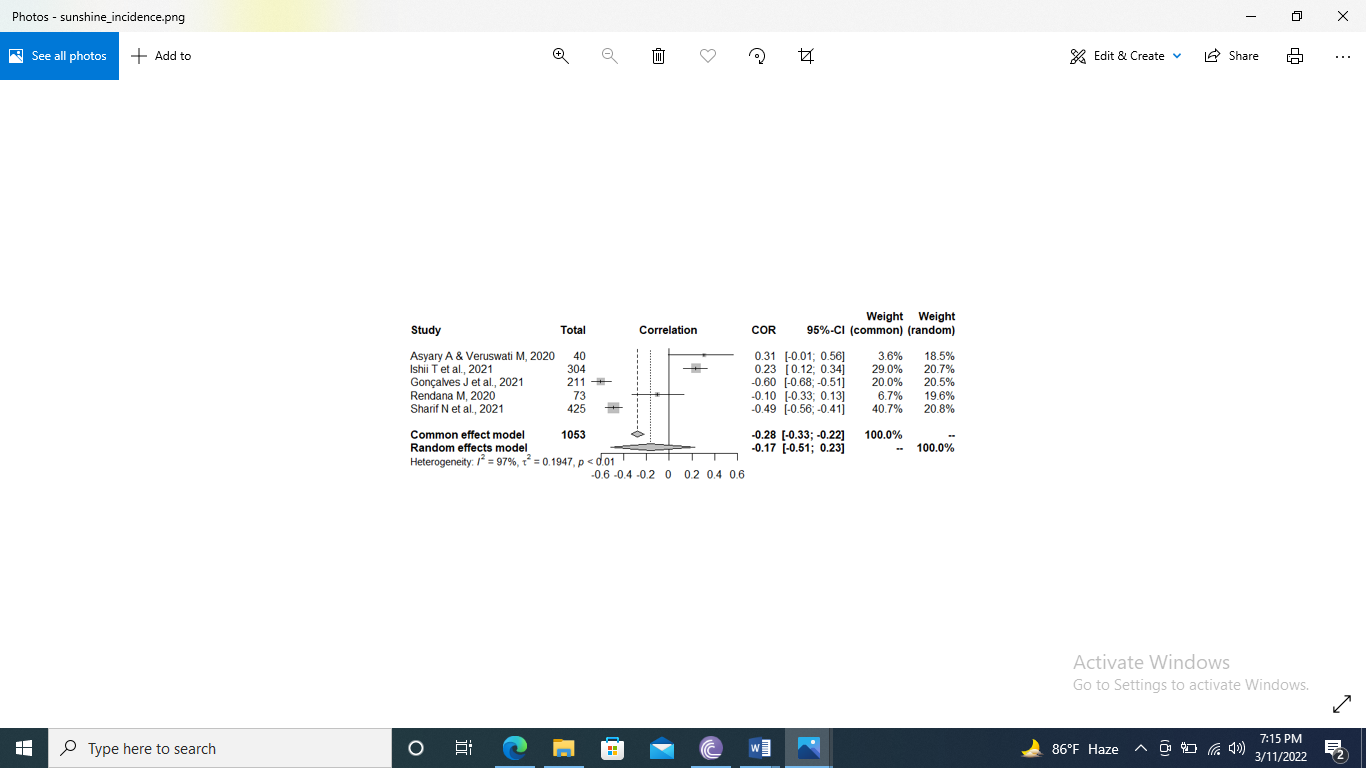


**Fig. S12**: Forest plot of COVID-19 incidence and sunlight.


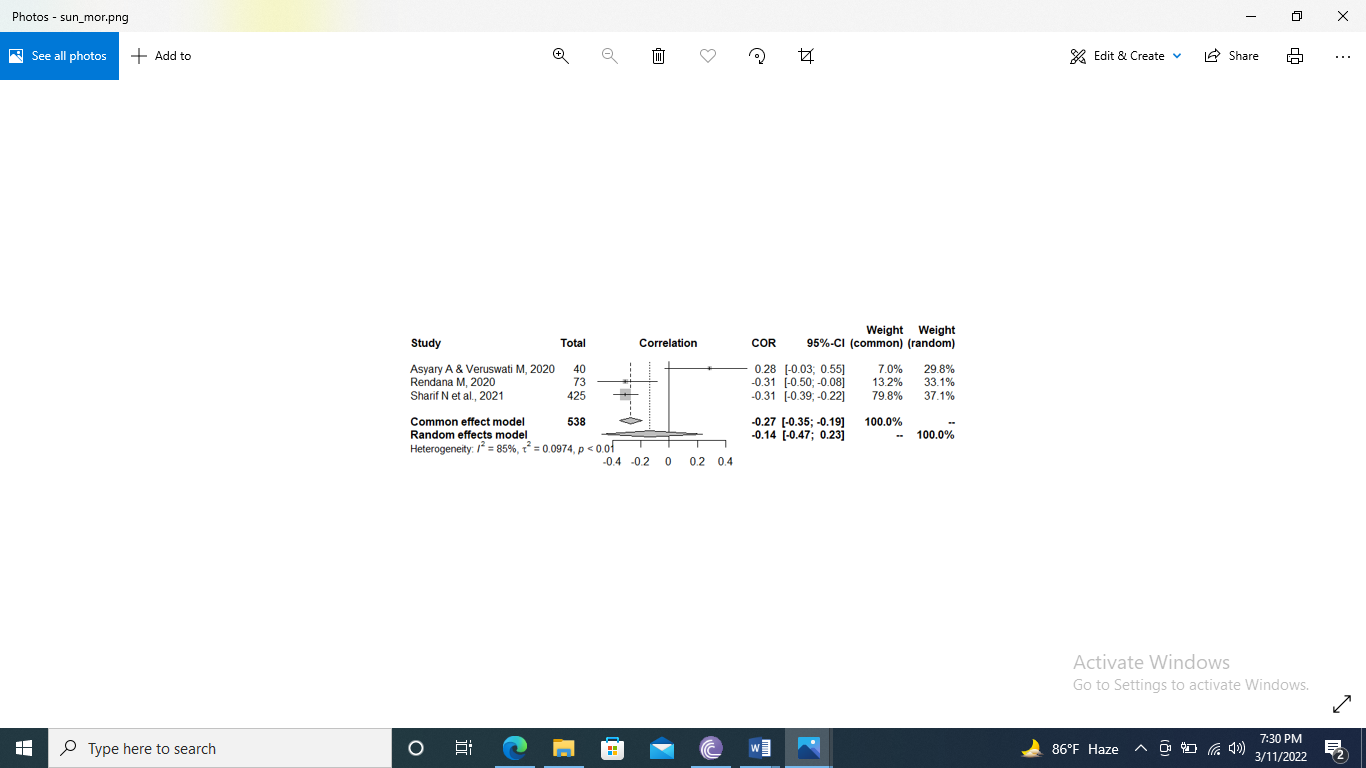


**Fig. S13**: Forest plot of COVID-19 deaths and sunlight.


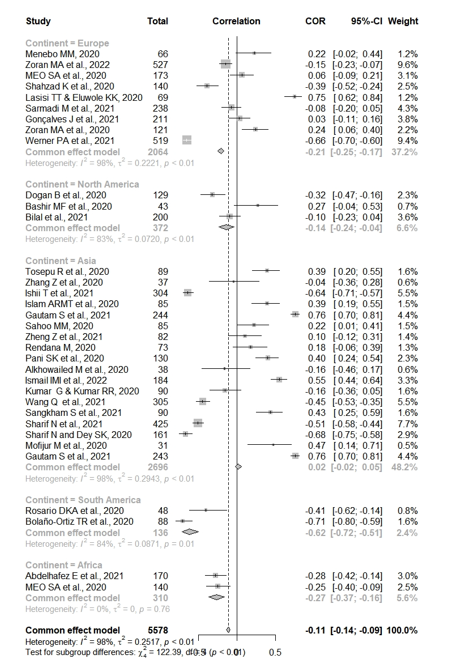


**Fig. S14**: Forest plot of subgroup analysis of COVID-19 incidence and temperature.


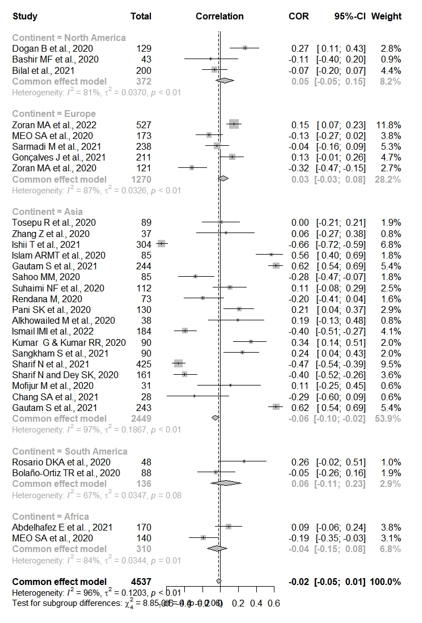


**Fig. S15**: Forest plot of subgroup analysis of COVID-19 incidence and relative humidity.


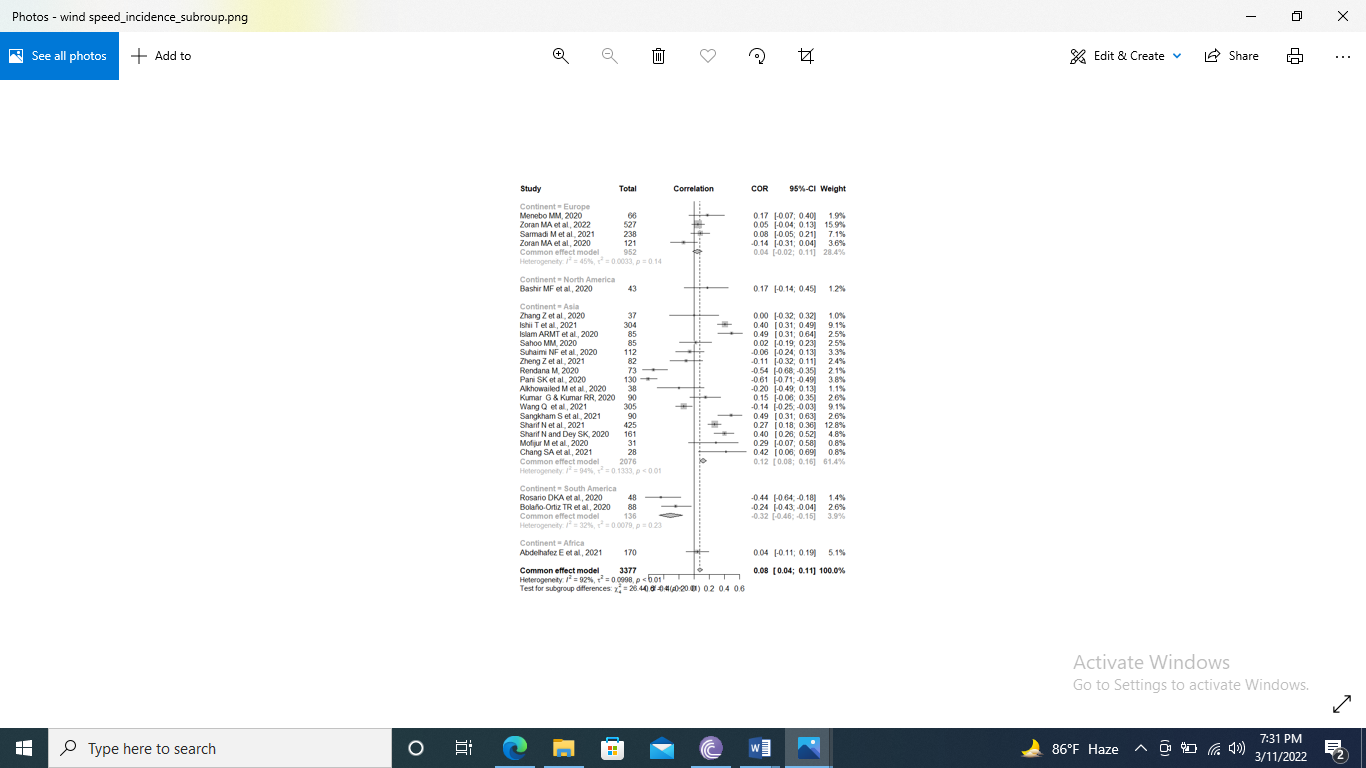


**Fig. S16**: Forest plot of subgroup analysis of COVID-19 incidence and wind speed.


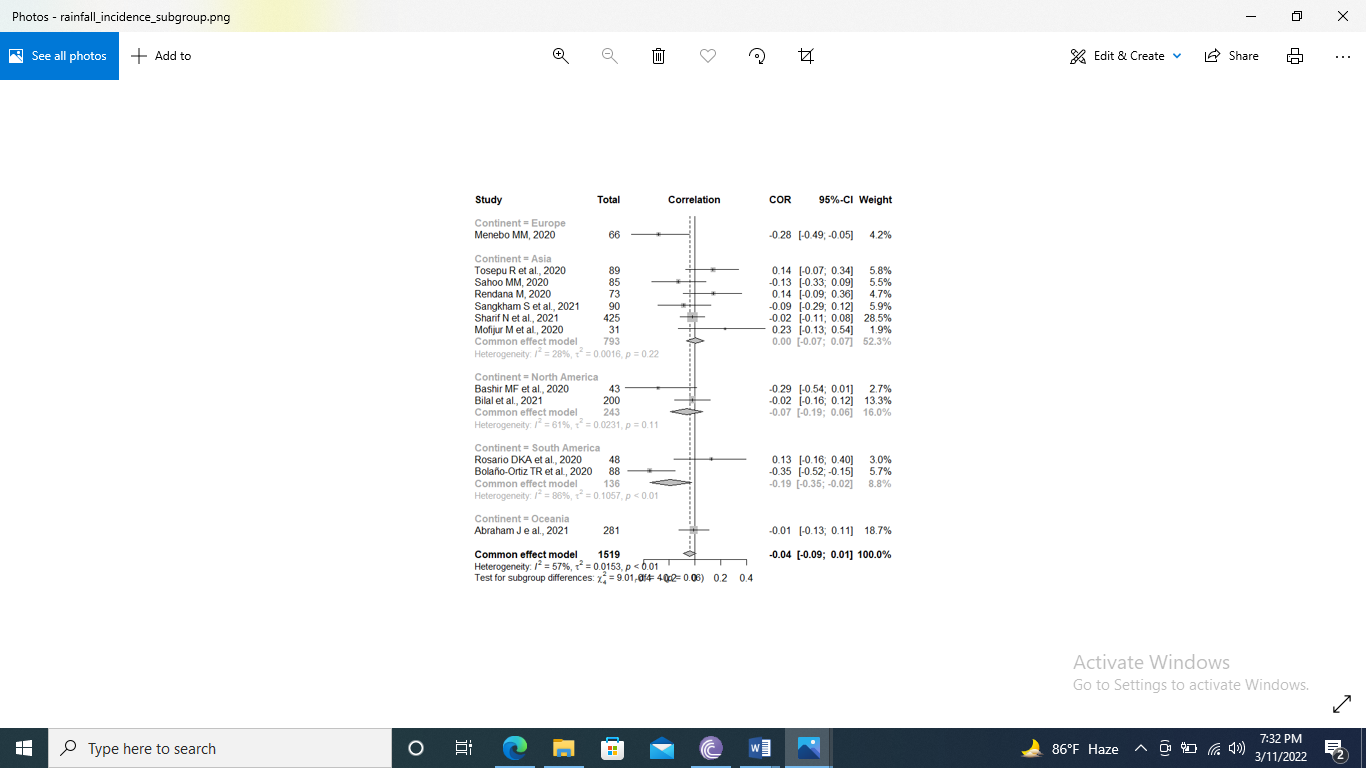


**Fig. S17**: Forest plot of subgroup analysis of COVID-19 incidence and rainfall.


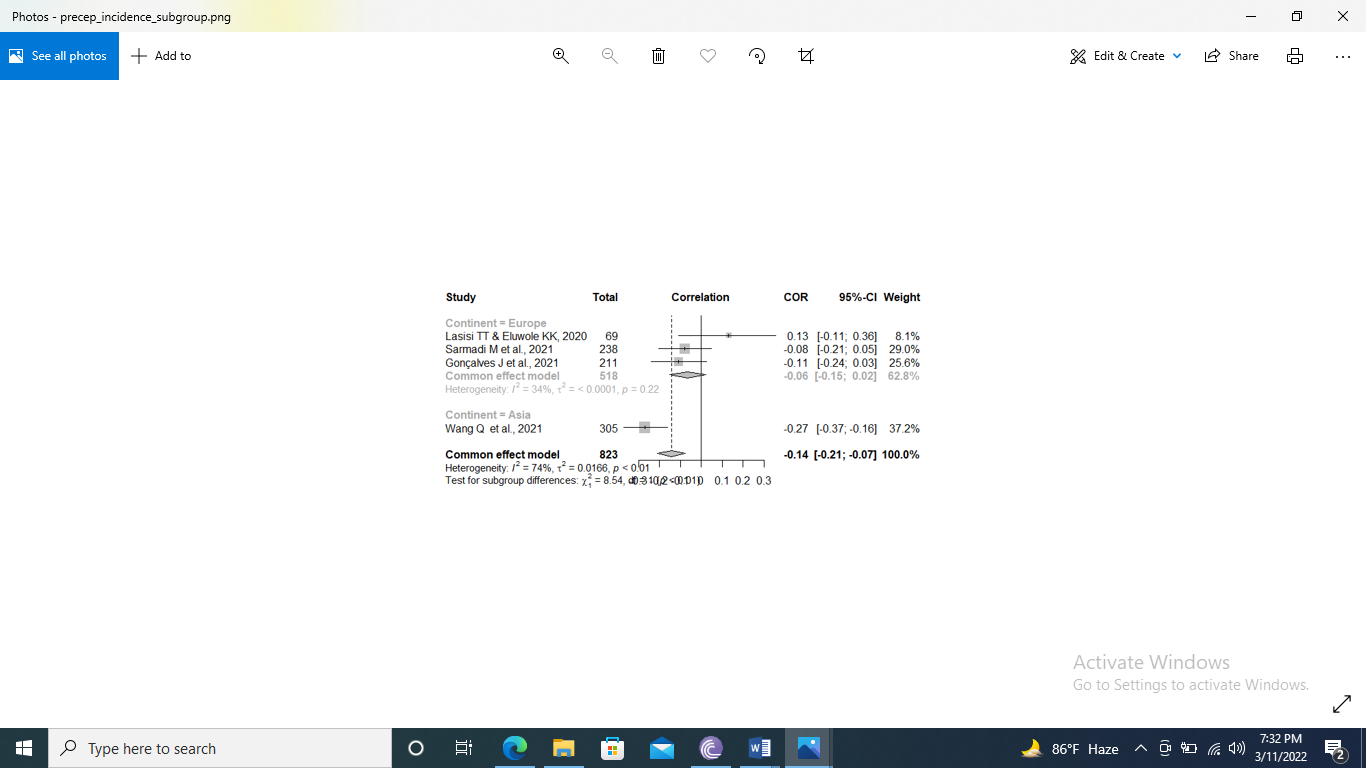


**Fig. S18**: Forest plot of subgroup analysis of COVID-19 incidence and precipitation.


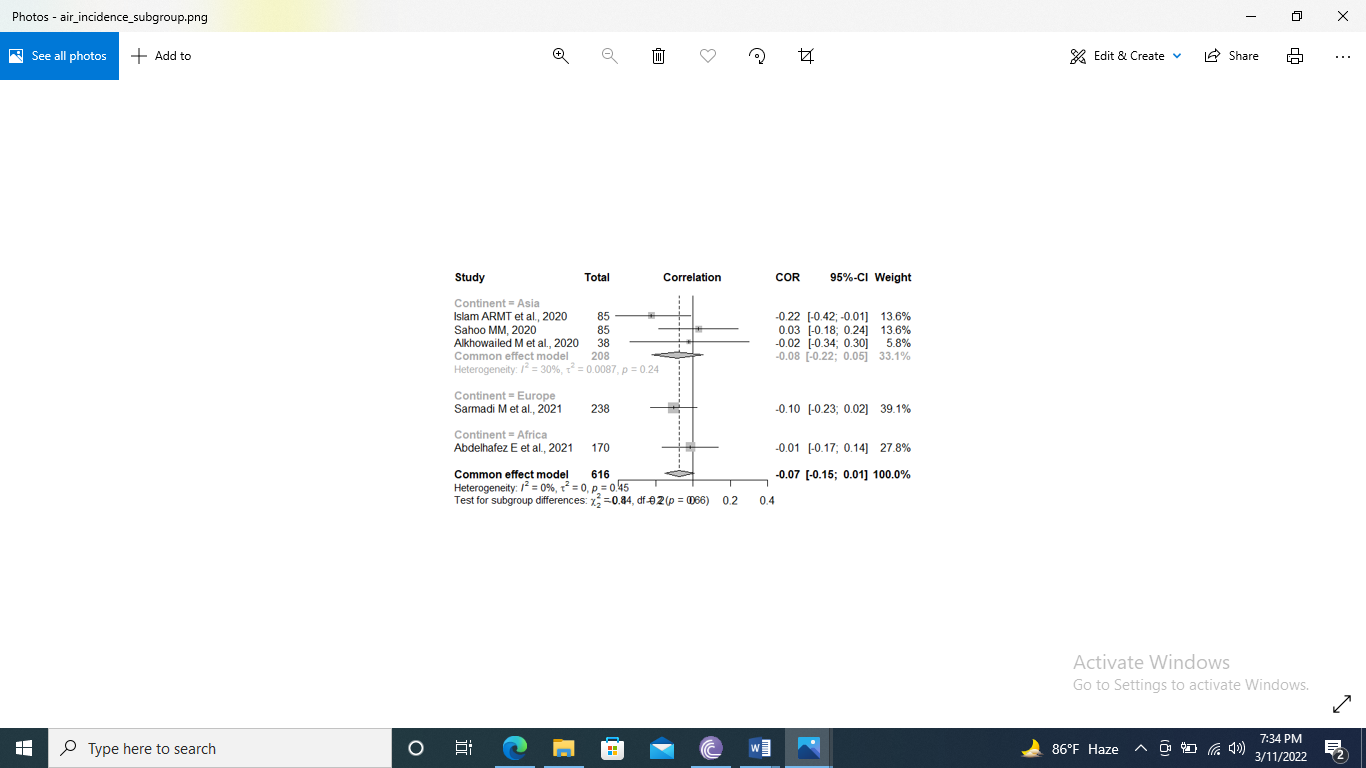


**Fig. S19**: Forest plot of subgroup analysis of COVID-19 incidence and air pressure.


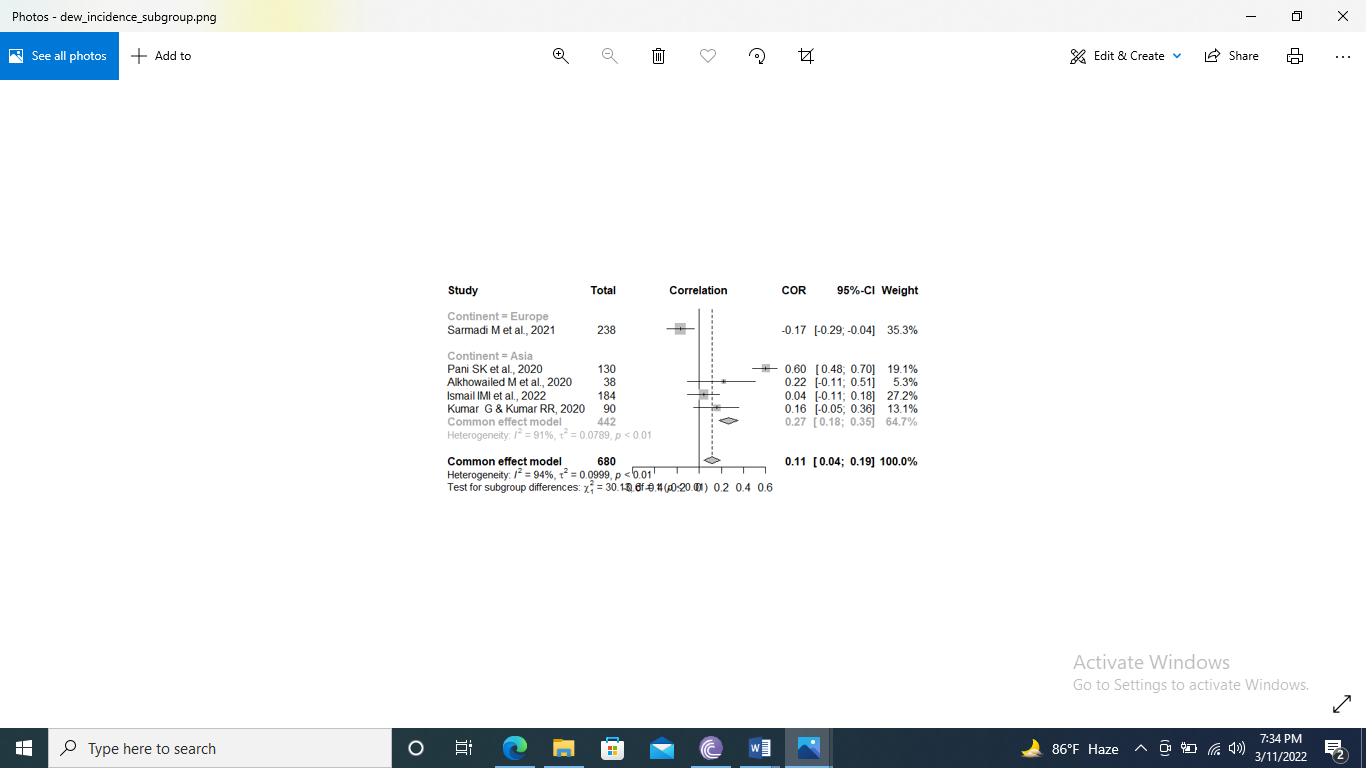


**Fig. S20**: Forest plot of subgroup analysis of COVID-19 incidence and dew point.


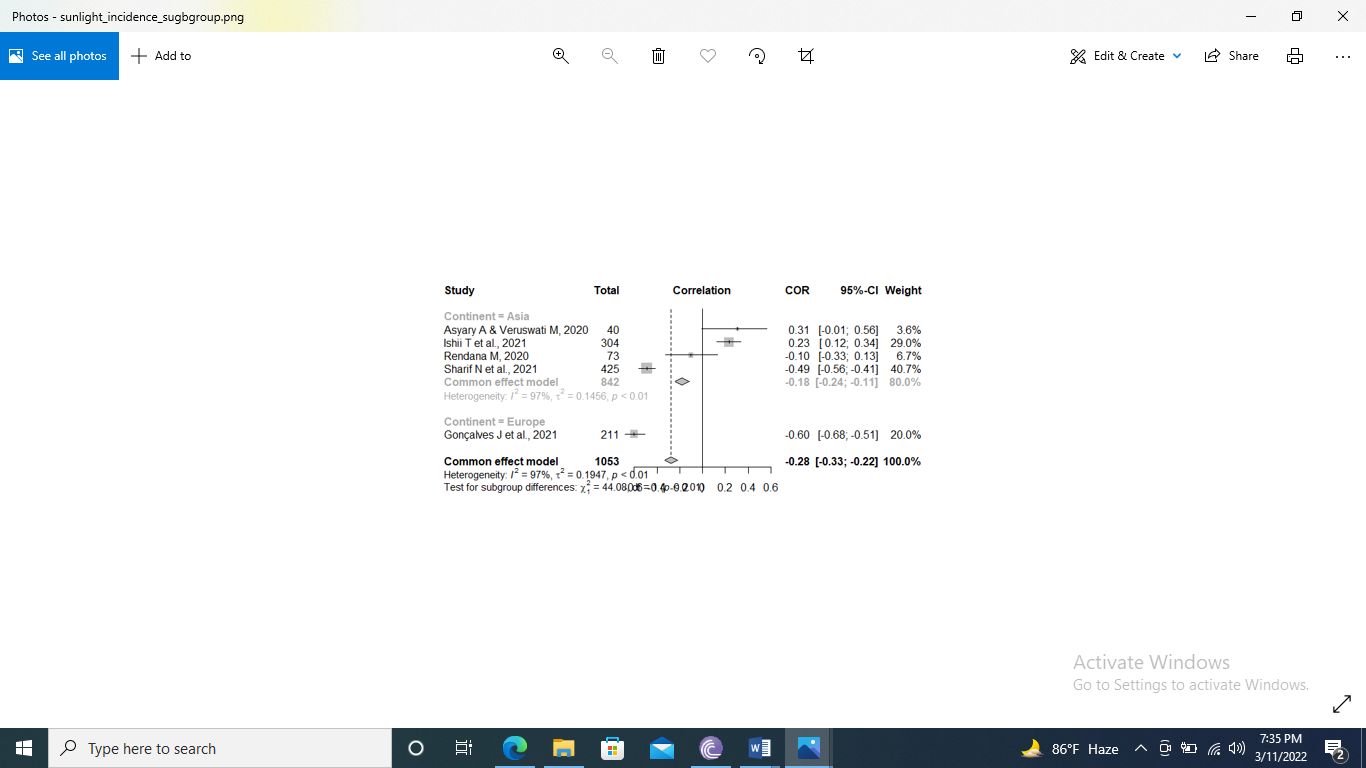


**Fig. S21**: Forest plot of subgroup analysis of COVID-19 incidence and sunlight.


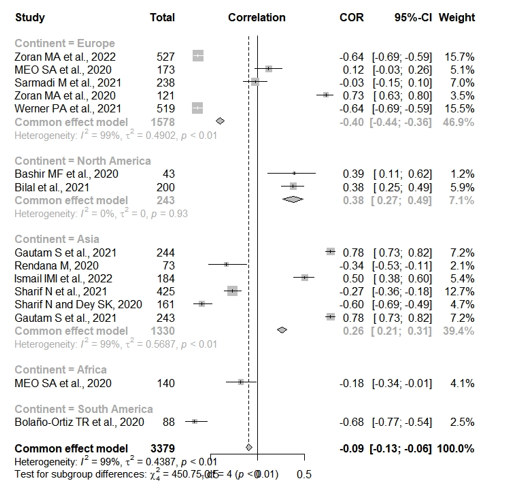


**Fig. S22**: Forest plot of subgroup analysis of COVID-19 deaths and temperature.


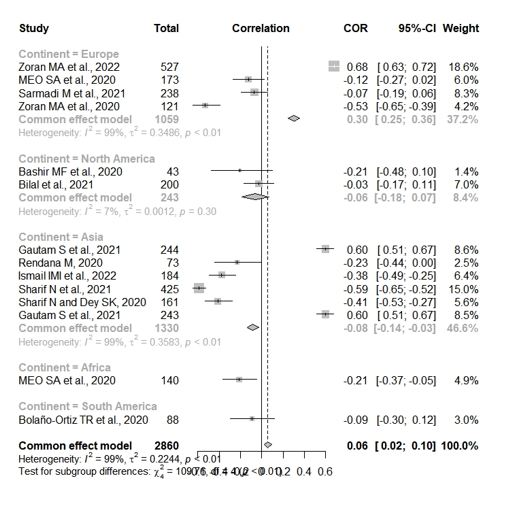


**Fig. S23**: Forest plot of subgroup analysis of COVID-19 deaths and relative humidity.


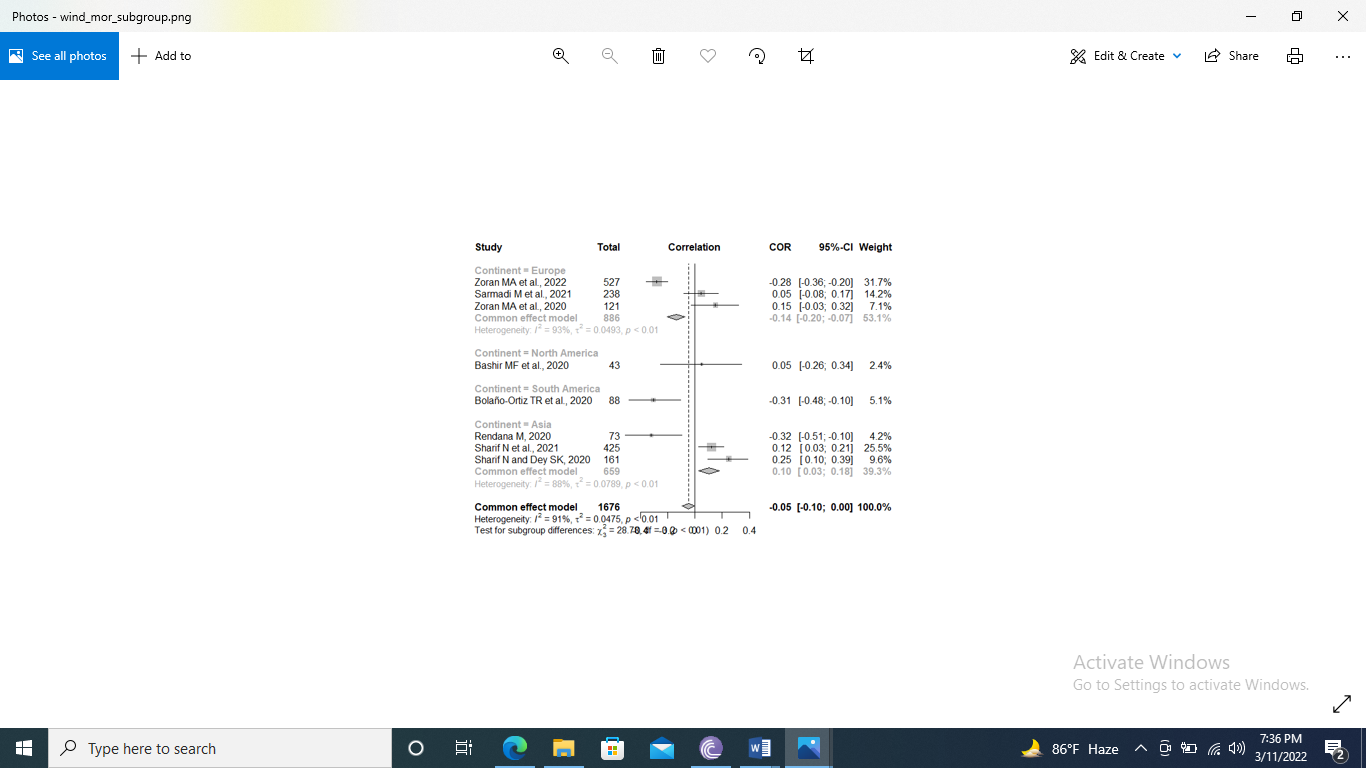


**Fig. S24**: Forest plot of subgroup analysis of COVID-19 deaths and wind speed.


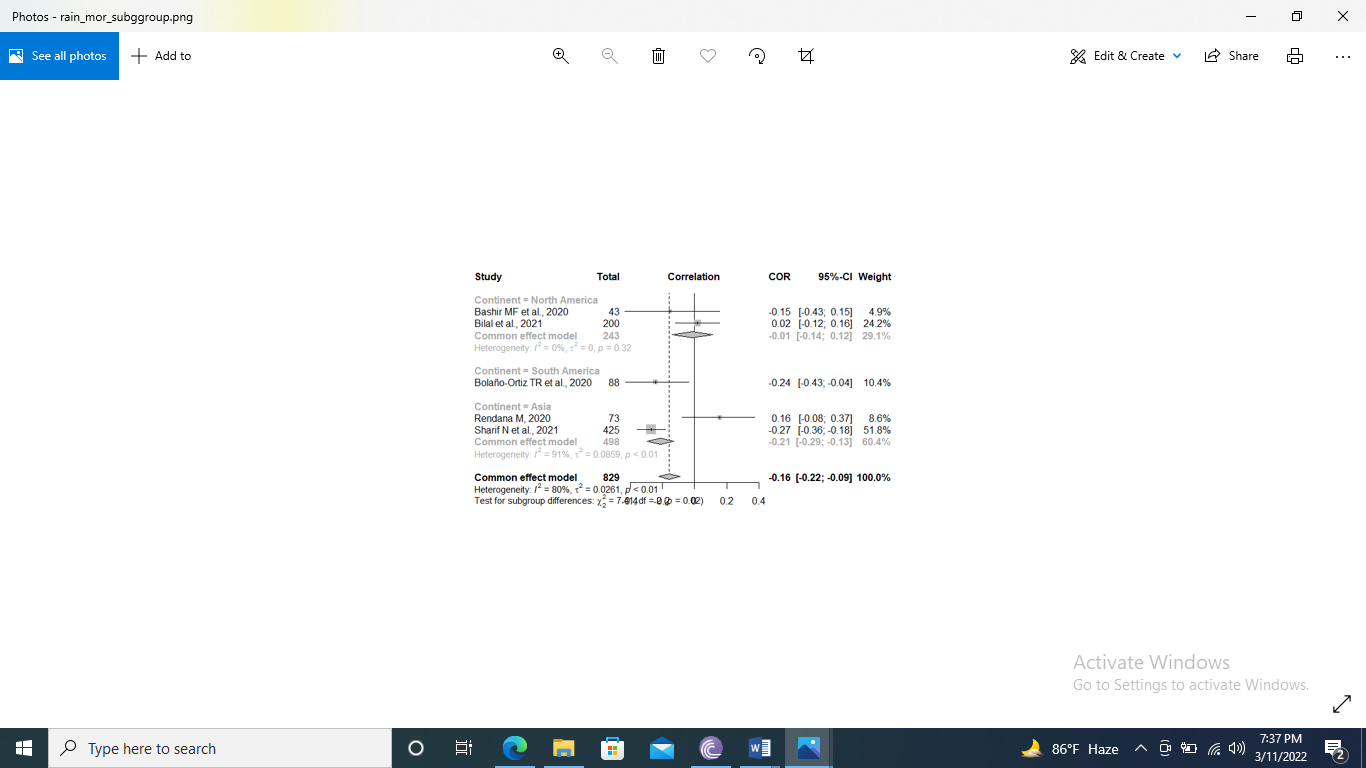


**Fig. S25**: Forest plot of subgroup analysis of COVID-19 deaths and rainfall.


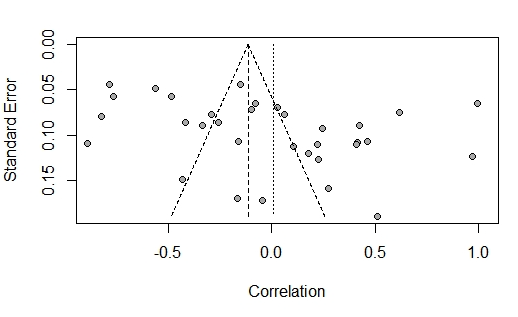


**Fig. S26**: Funnel plot of COVID-19 incidence and temperature.


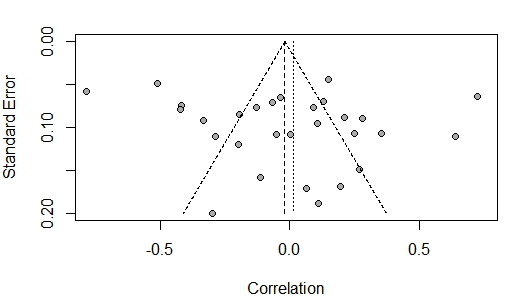


**Fig. S27**: Funnel plot of COVID-19 incidence and relative humidity.


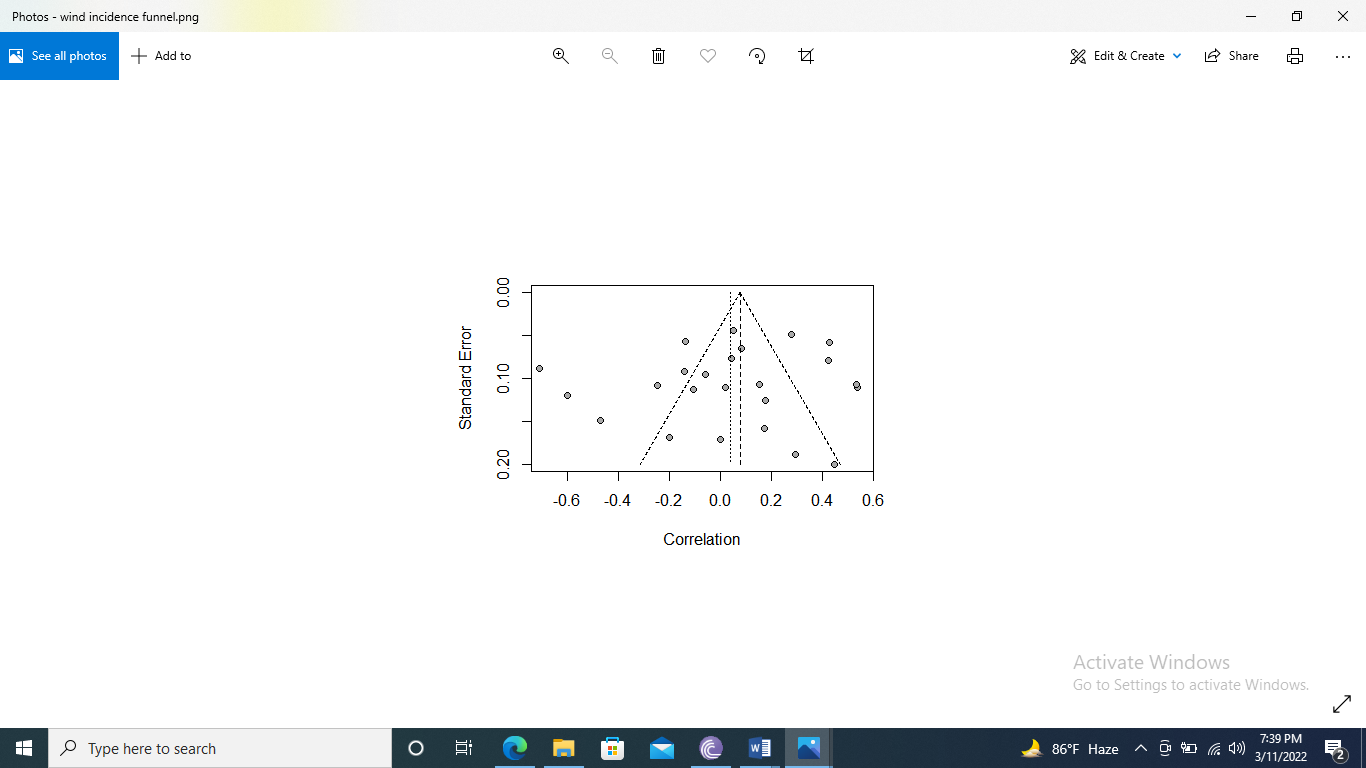


**Fig. S28**: Funnel plot of COVID-19 incidence and wind speed.


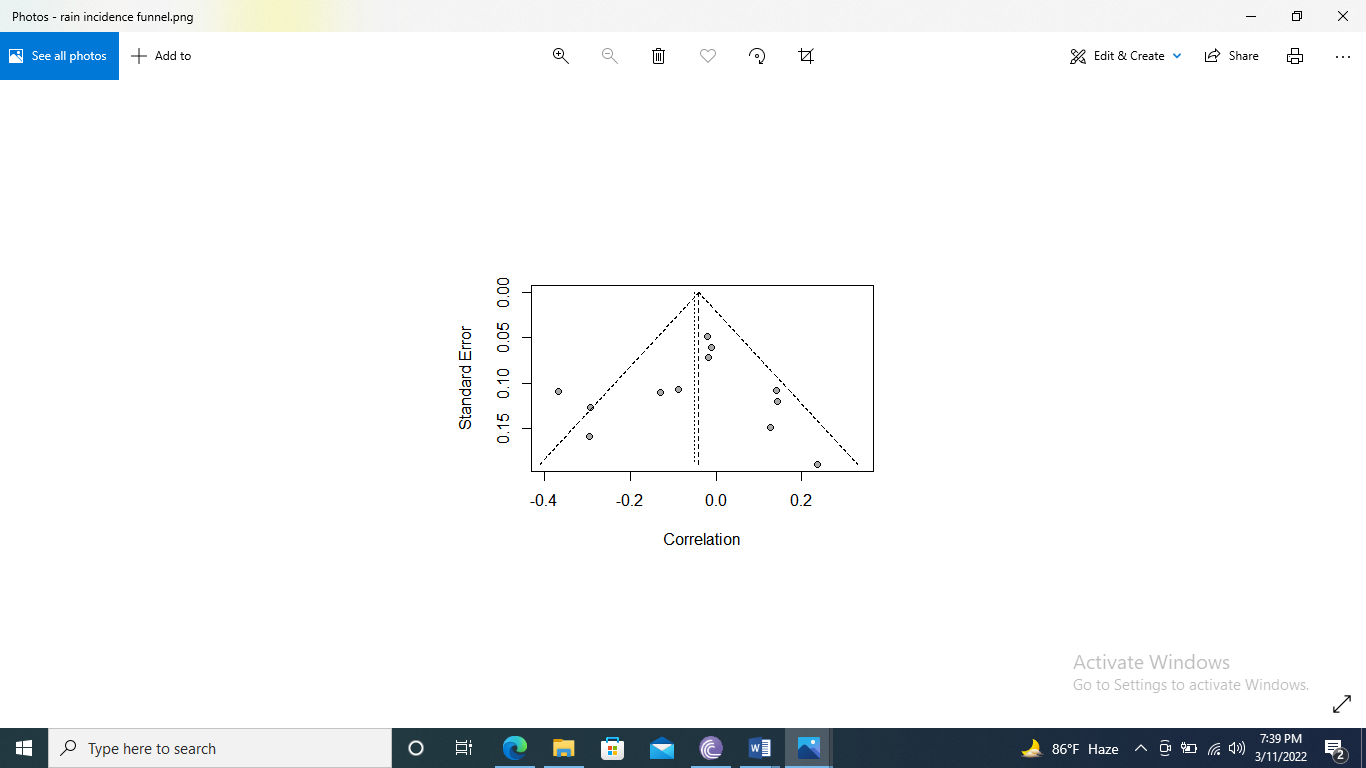


**Fig. S29**: Funnel plot of COVID-19 incidence and rainfall.


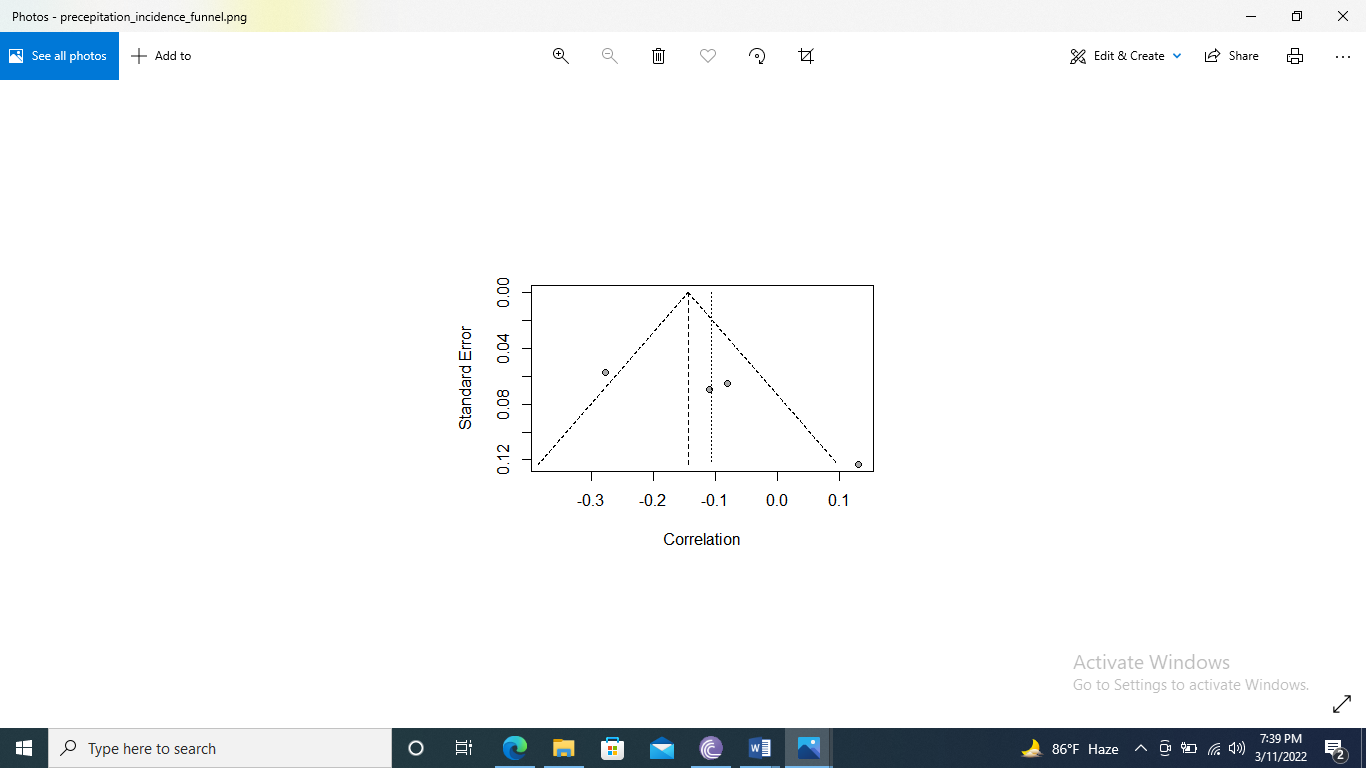


**Fig. S30**: Funnel plot of COVID-19 incidence and precipitation.


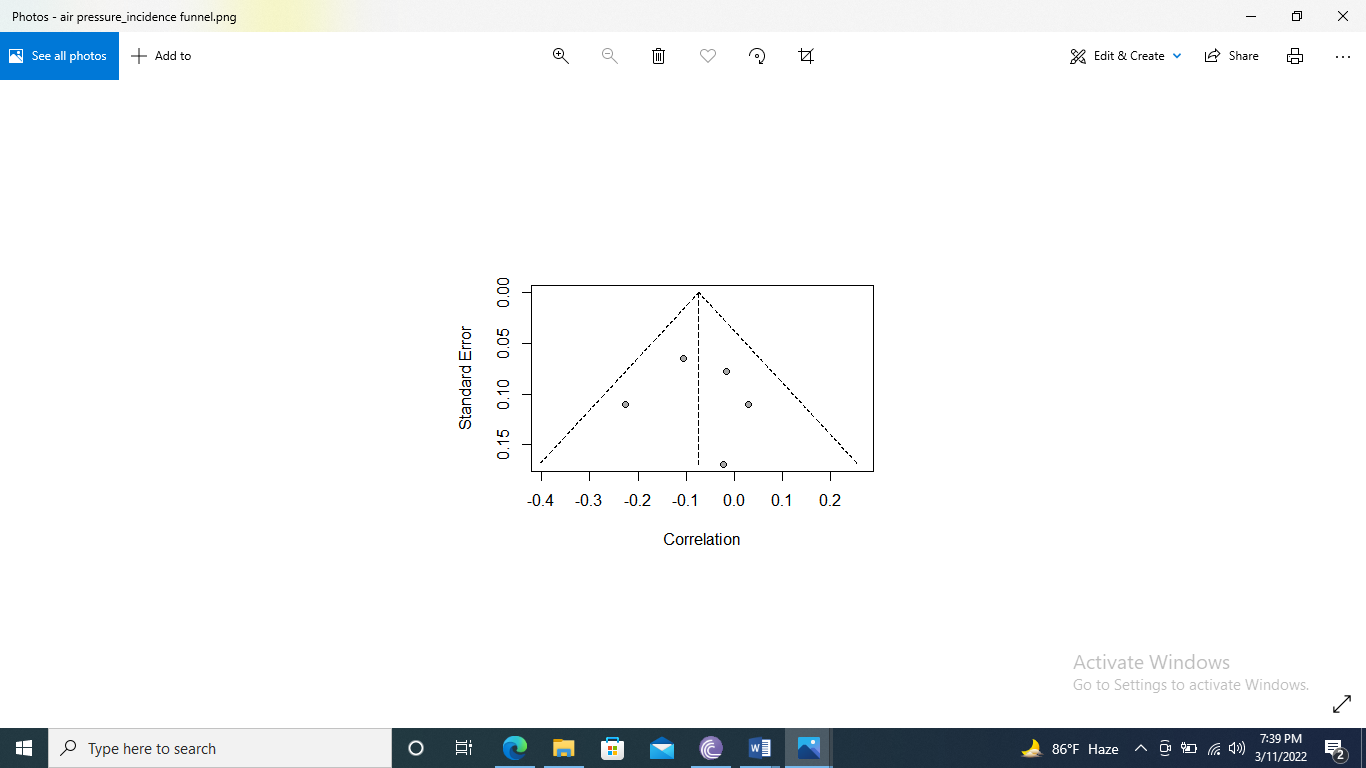


**Fig. S31**: Funnel plot of COVID-19 incidence and air pressure.


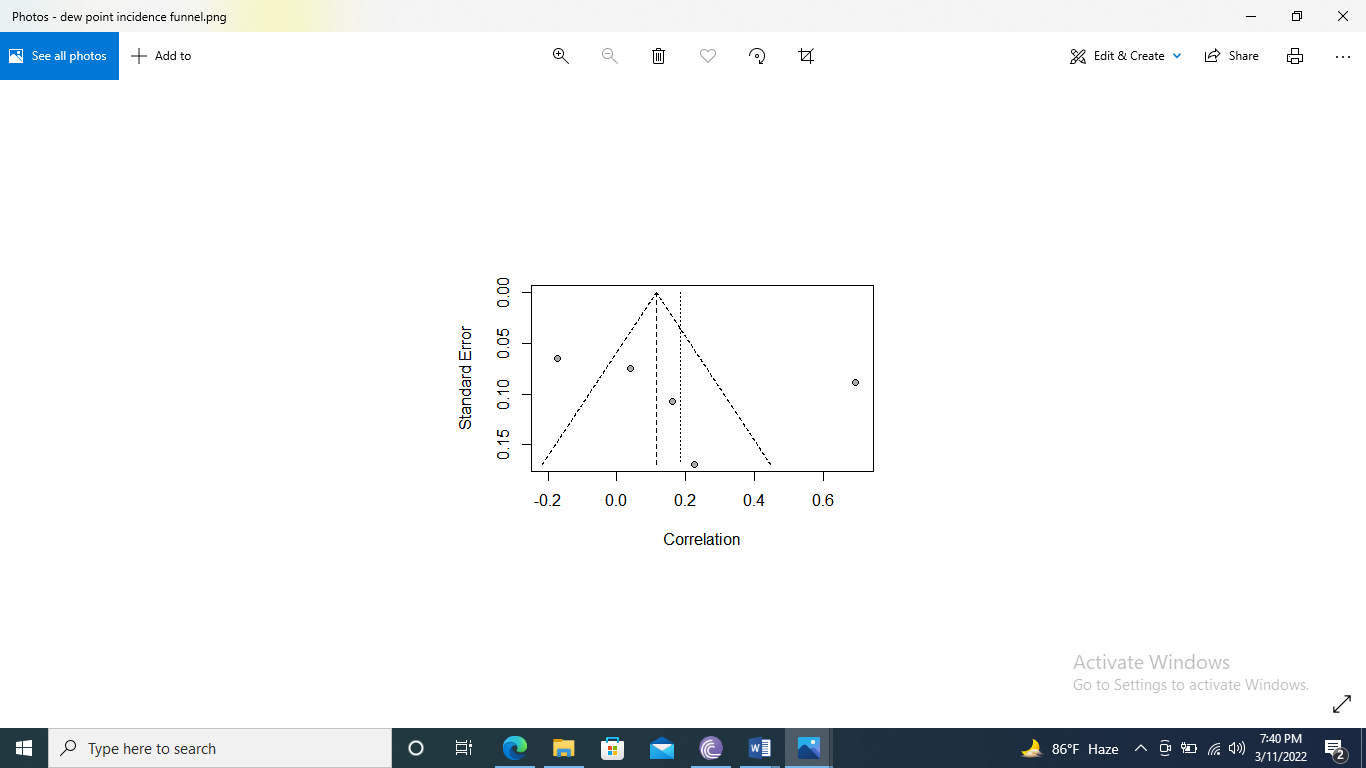


**Fig. S32**: Funnel plot of COVID-19 incidence and dew point.


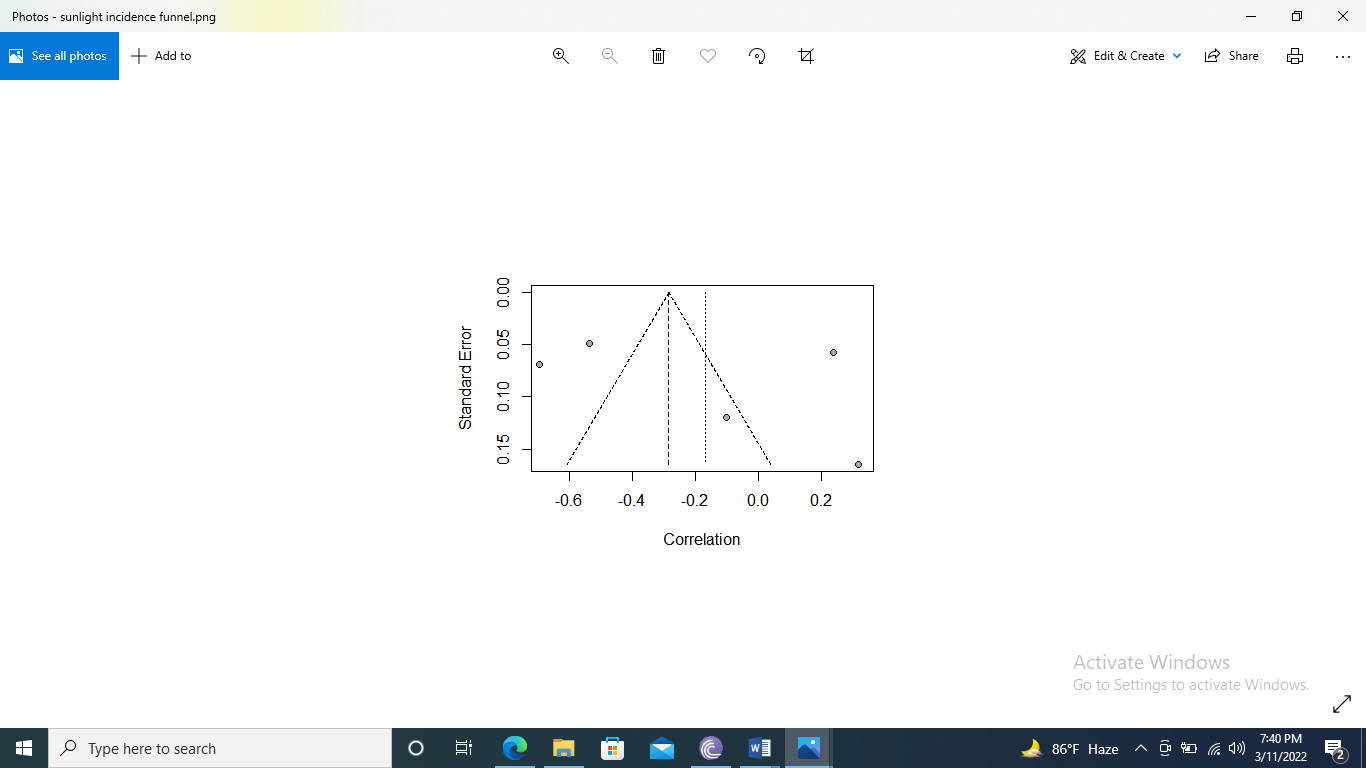


**Fig. S33**: Funnel plot of COVID-19 incidence and sunlight.


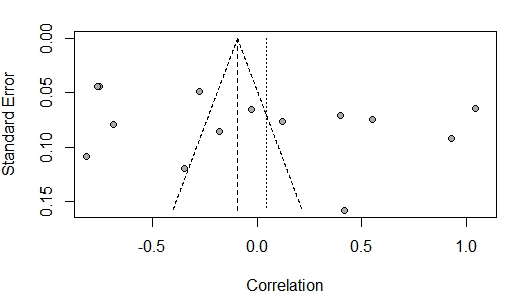


**Fig. S34**: Funnel plot of COVID-19 deaths and temperature.


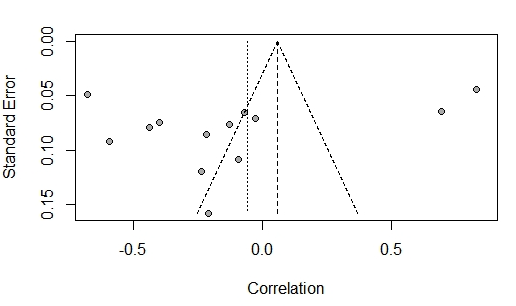


**Fig. S35**: Funnel plot of COVID-19 deaths and relative humidity.


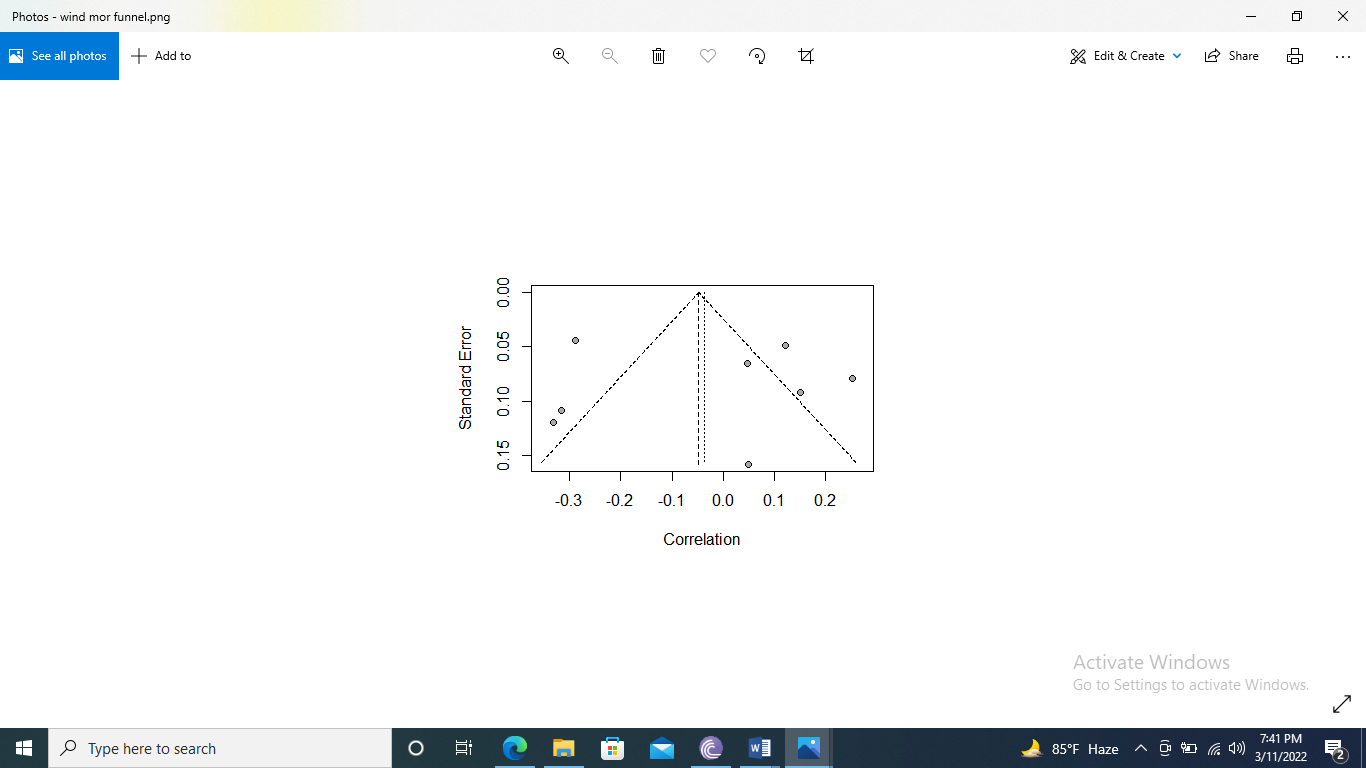


**Fig. S36**: Funnel plot of COVID-19 deaths and wind speed.


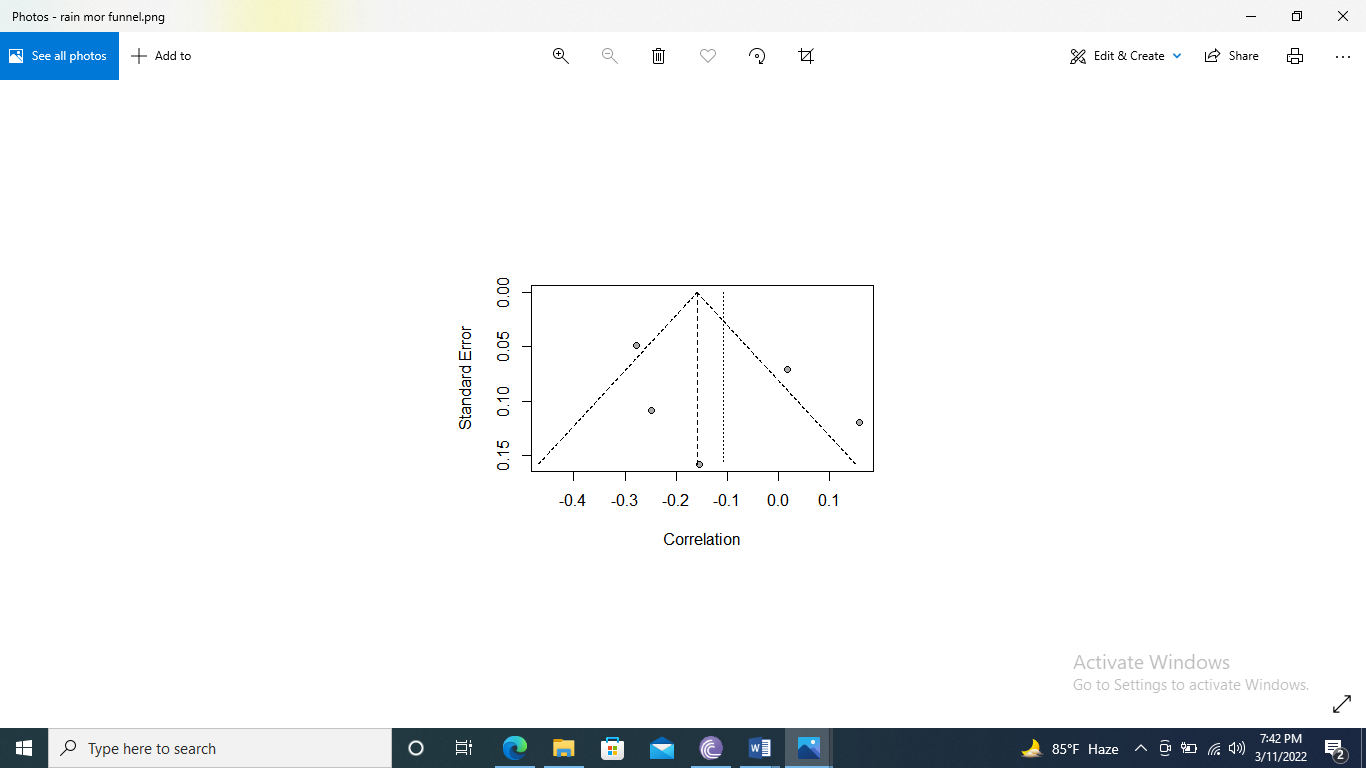


**Fig. S37**: Funnel plot of COVID-19 deaths and rainfall.


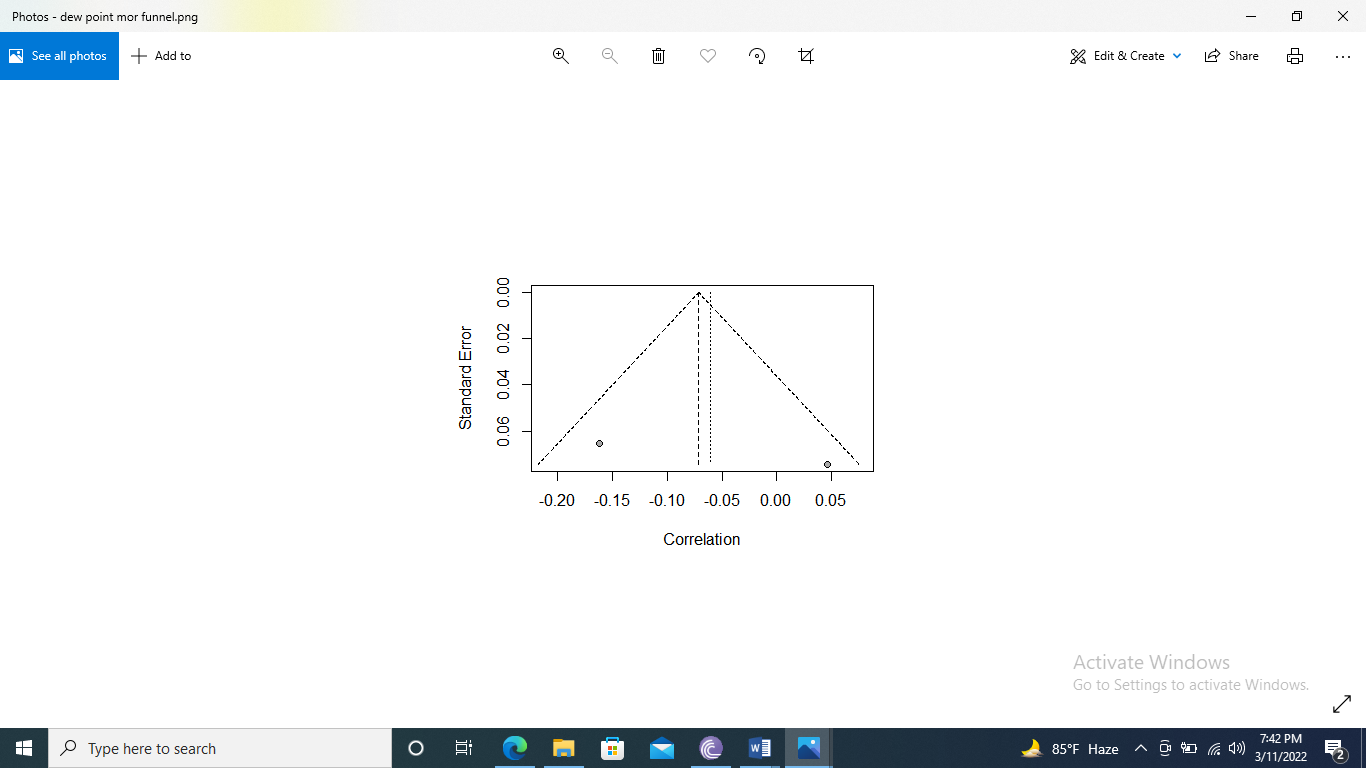


**Fig. S38**: Funnel plot of COVID-19 deaths and dew point.


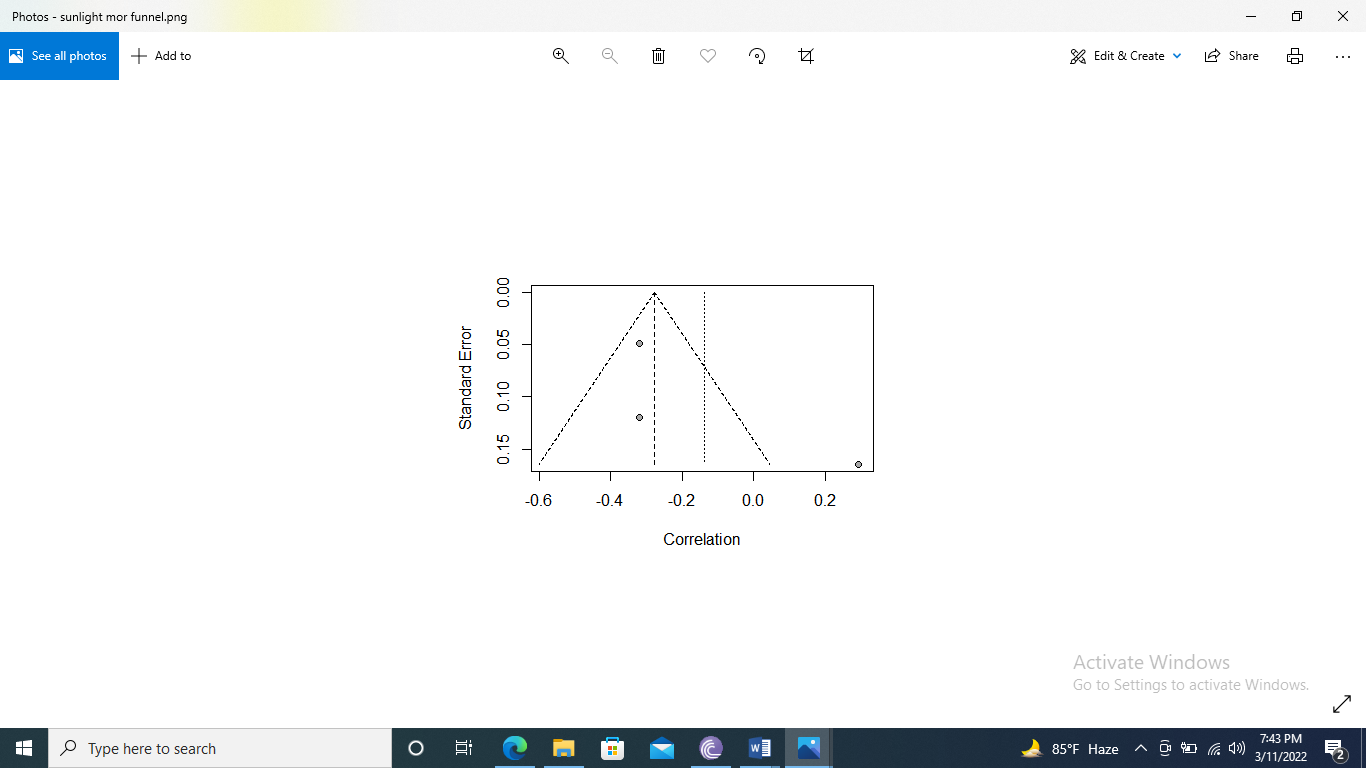


**Fig. S39**: Funnel plot of COVID-19 deaths and sunlight.


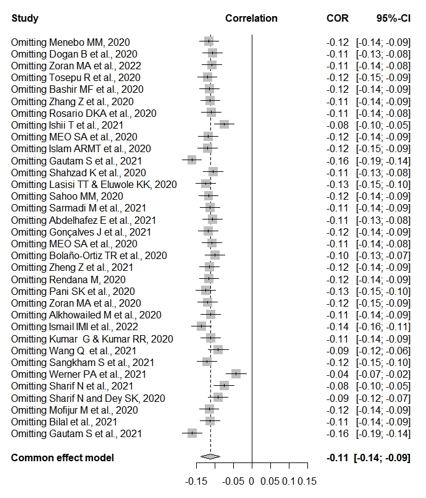


**Fig. S40**: Forest plot of sensitivity analysis of COVID-19 incidence and temperature.


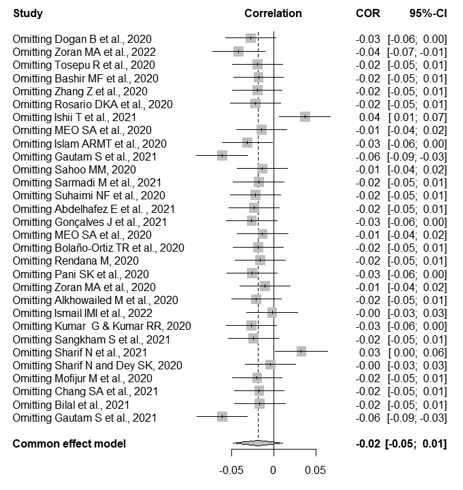


**Fig. S41**: Forest plot of sensitivity analysis of COVID-19 incidence and relative humidity.


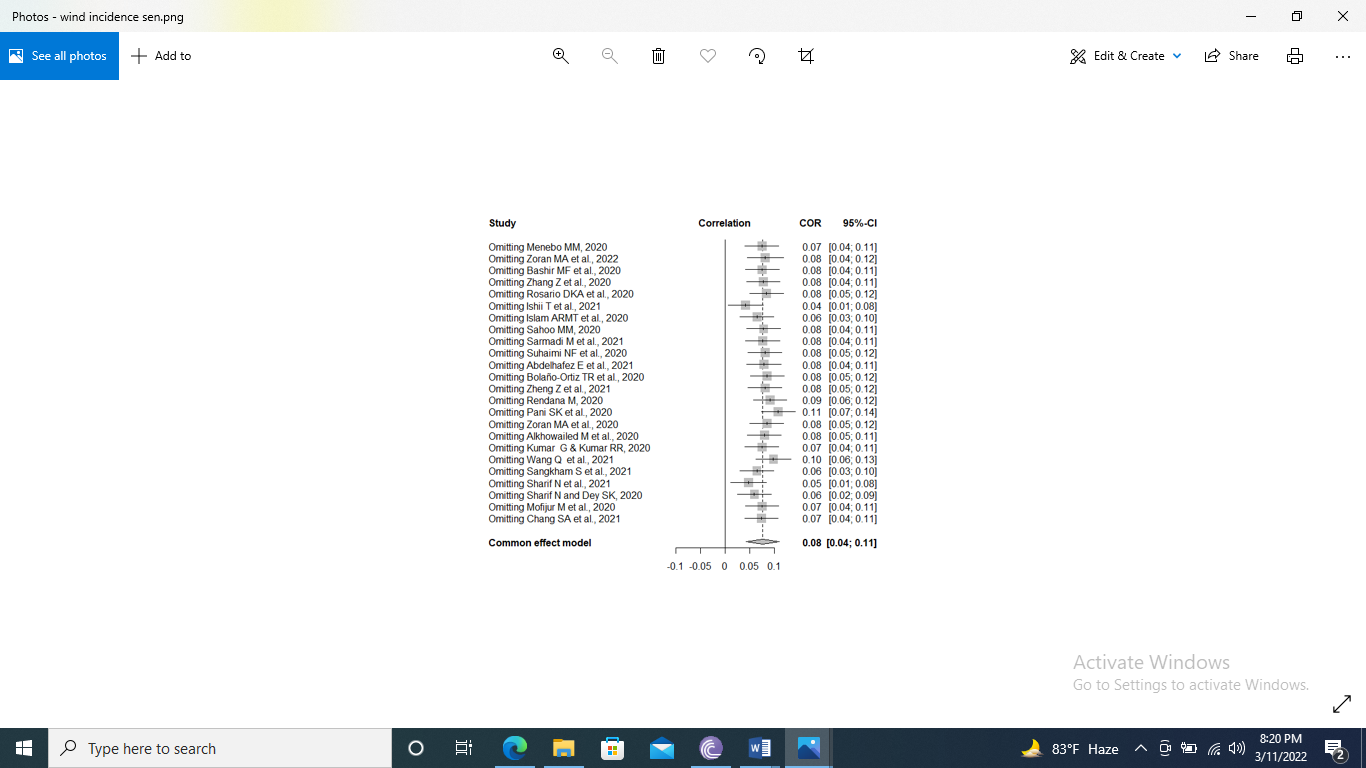


**Fig. S42**: Forest plot of sensitivity analysis of COVID-19 incidence and wind speed.


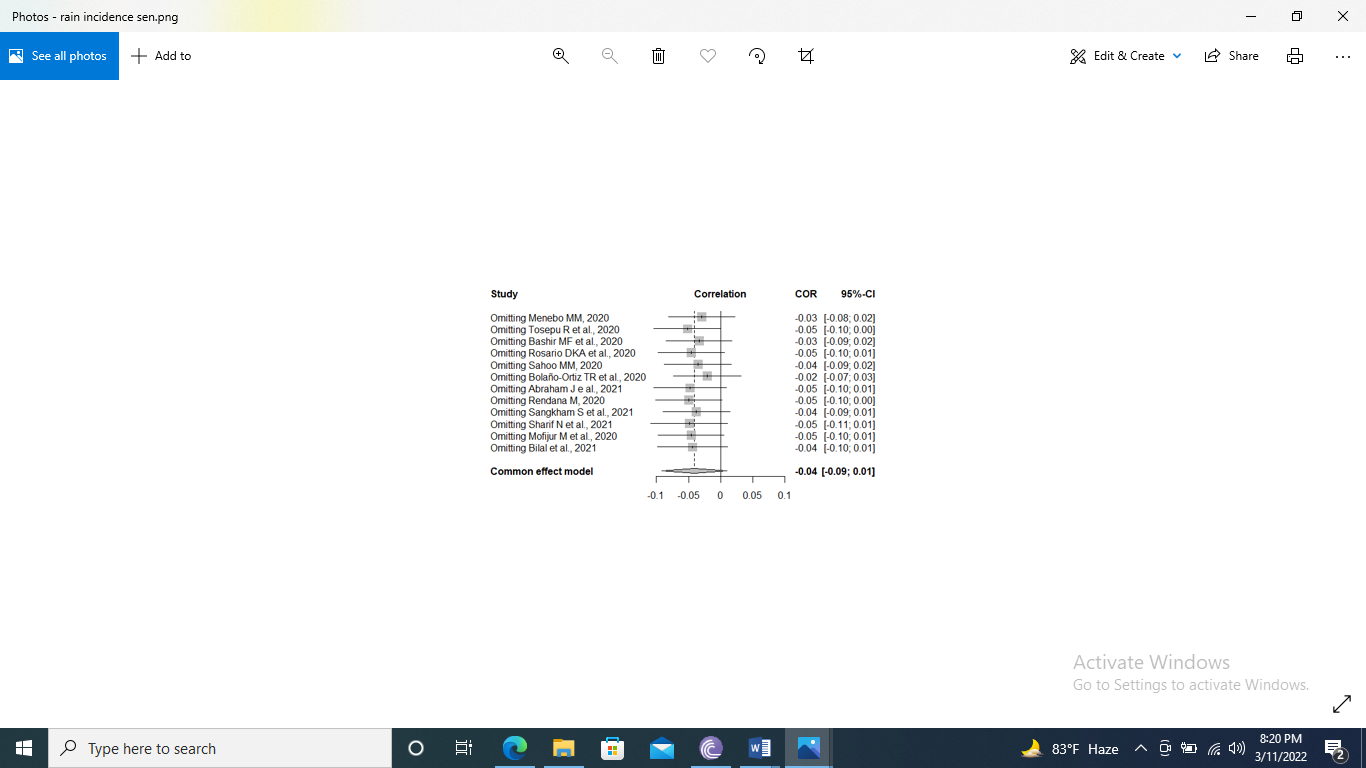


**Fig. S43**: Forest plot of sensitivity analysis of COVID-19 incidence and rainfall.


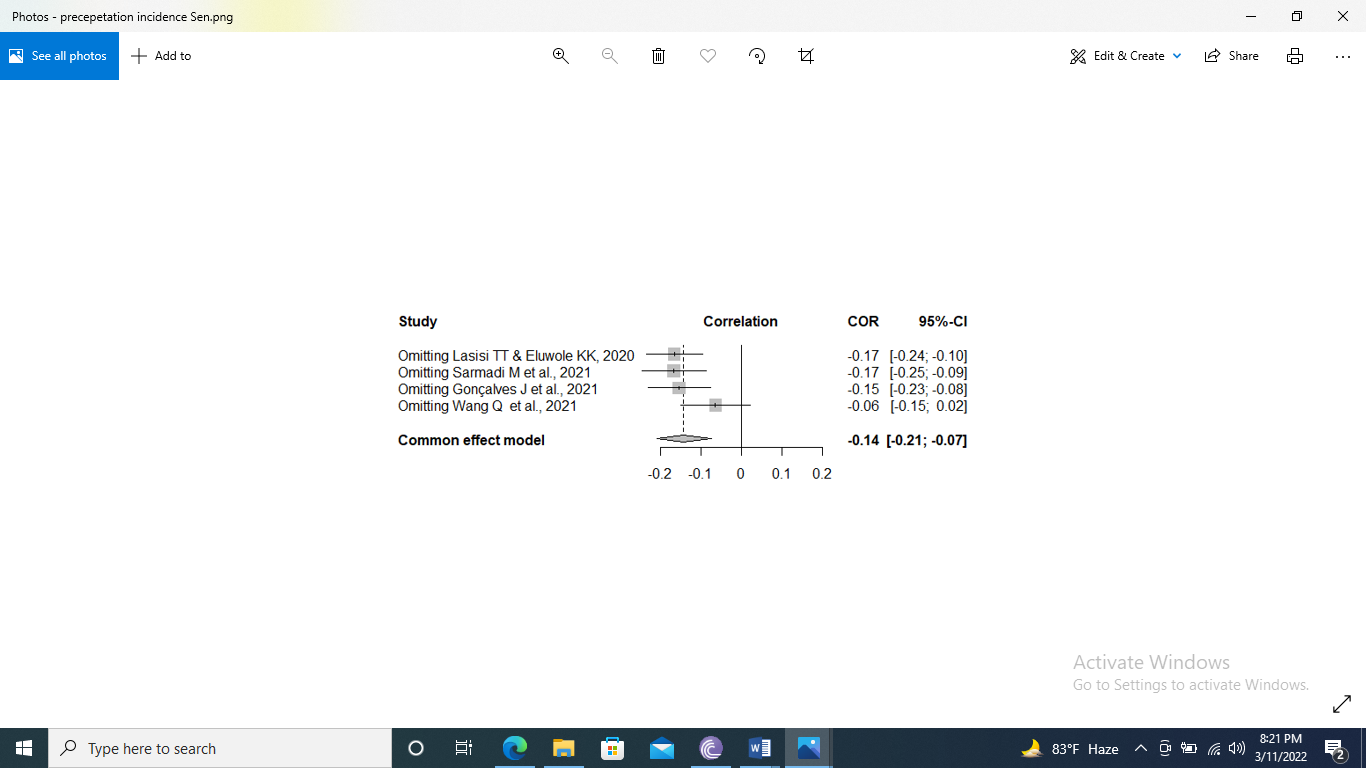


**Fig. S44**: Forest plot of sensitivity analysis of COVID-19 incidence and precipitation.


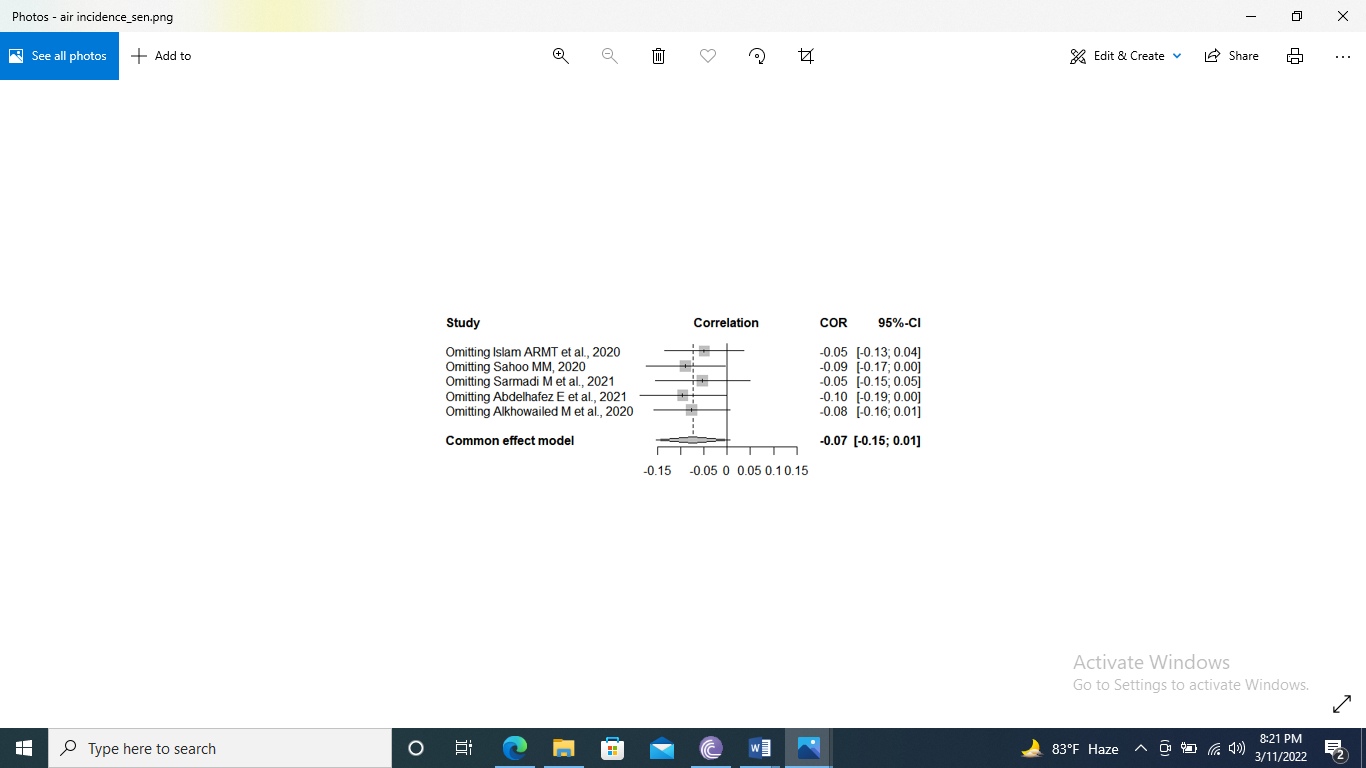


**Fig. S45**: Forest plot of sensitivity analysis of COVID-19 incidence and air pressure.


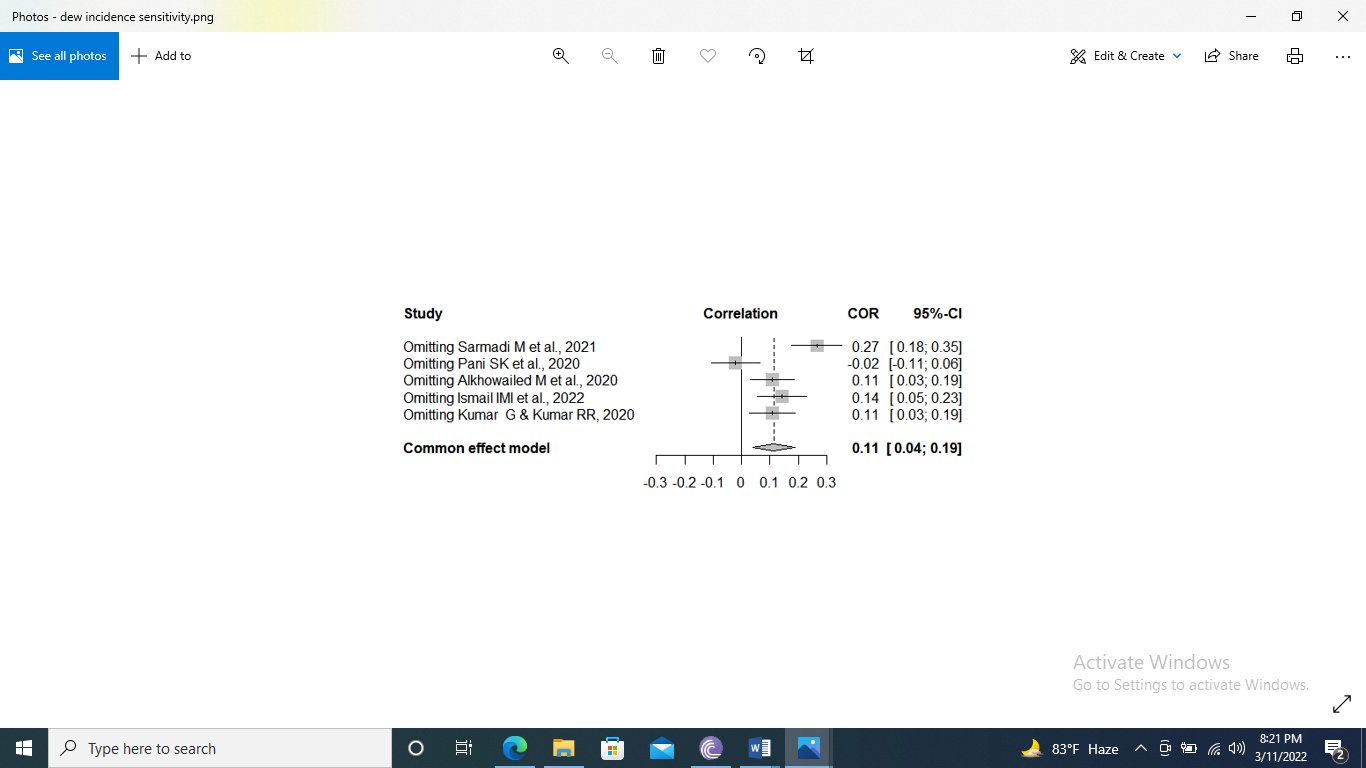


**Fig. S46**: Forest plot of sensitivity analysis of COVID-19 incidence and dew point.


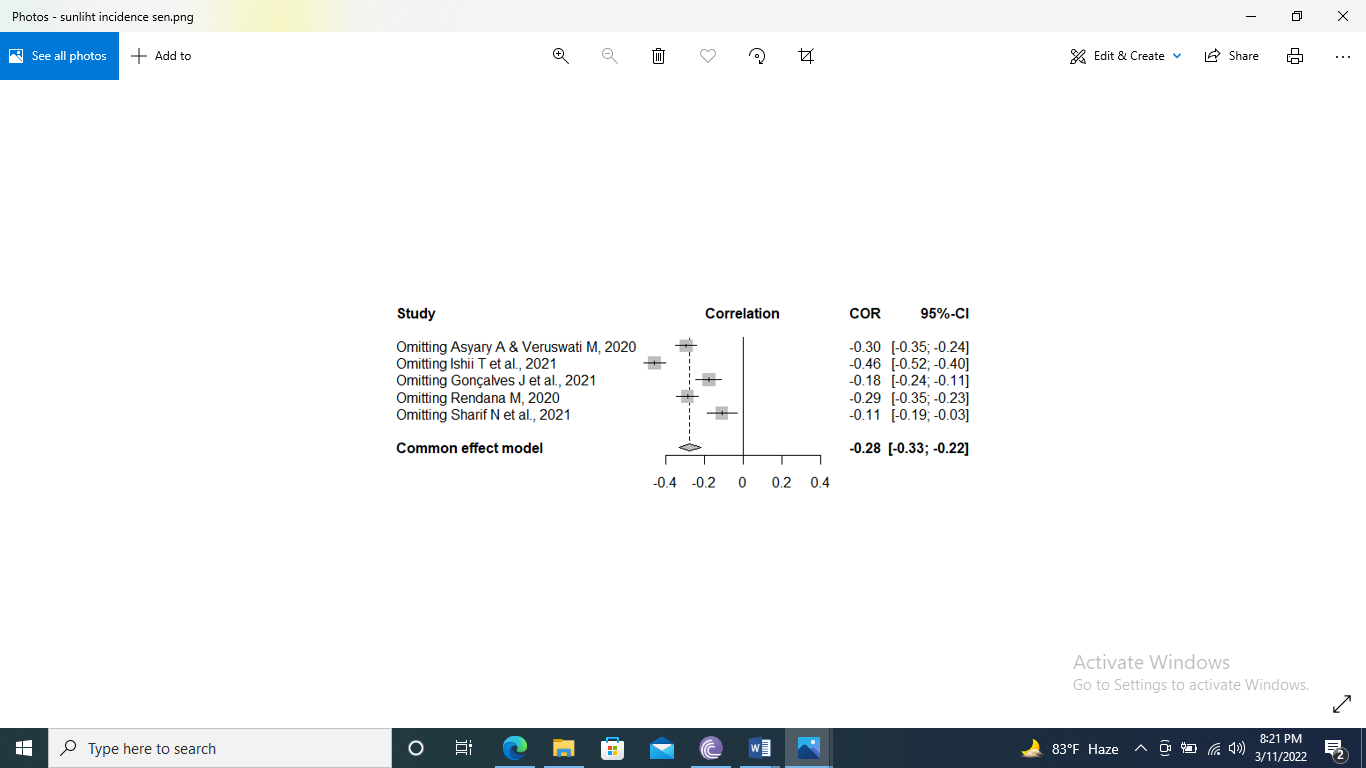


**Fig. S47**: Forest plot of sensitivity analysis of COVID-19 incidence and sunlight.


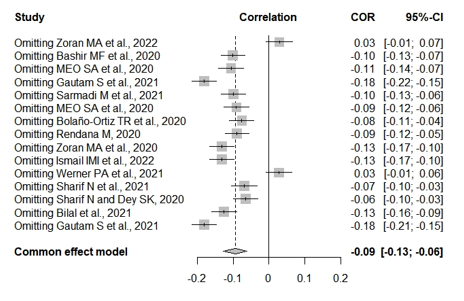


**Fig. S48**: Forest plot of sensitivity analysis of COVID-19 deaths and temperature.


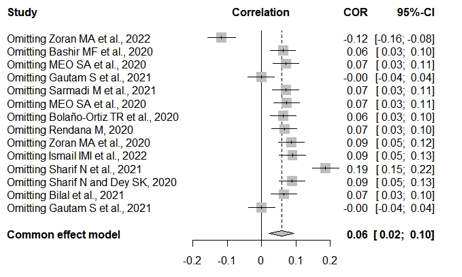


**Fig. S49**: Forest plot of sensitivity analysis of COVID-19 deaths and relative humidity.


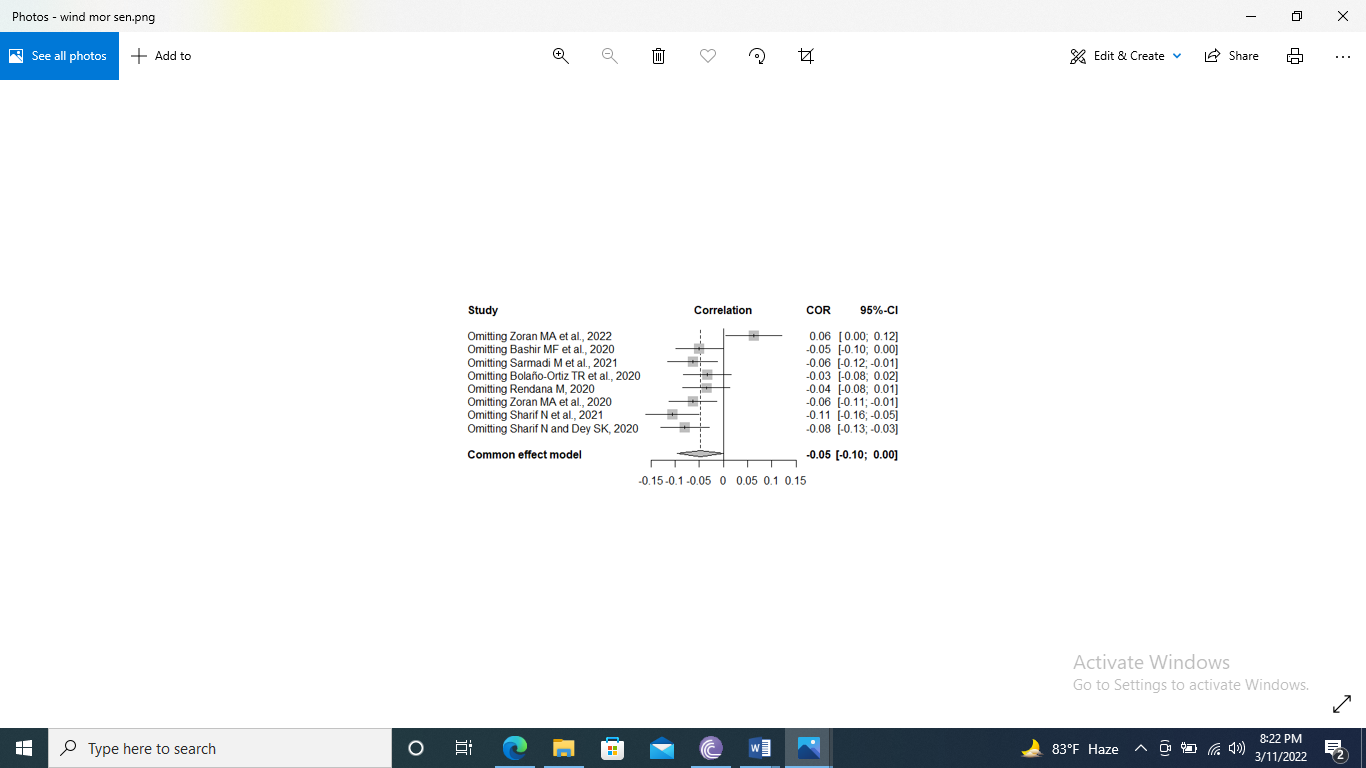


**Fig. S50**: Forest plot of sensitivity analysis of COVID-19 deaths and wind speed.


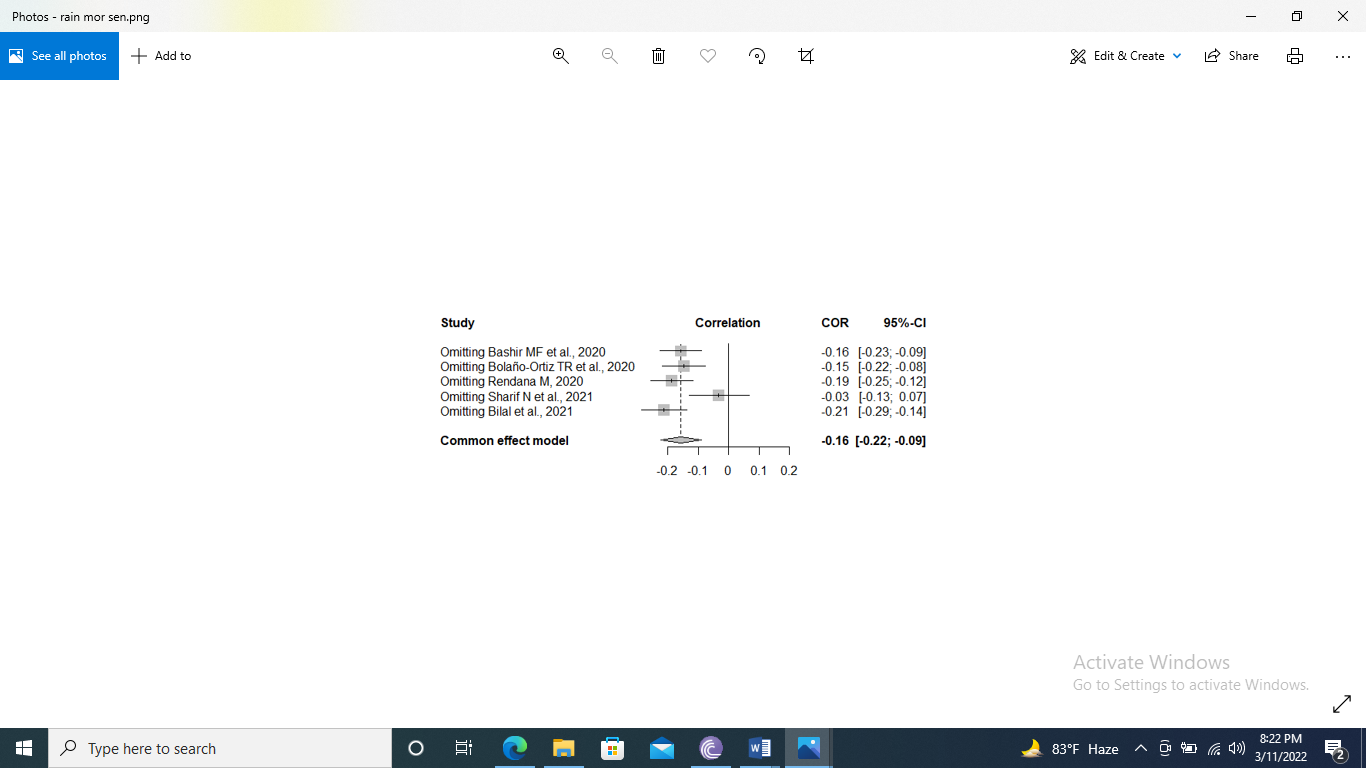


**Fig. S51**: Forest plot of sensitivity analysis of COVID-19 deaths and rainfall.


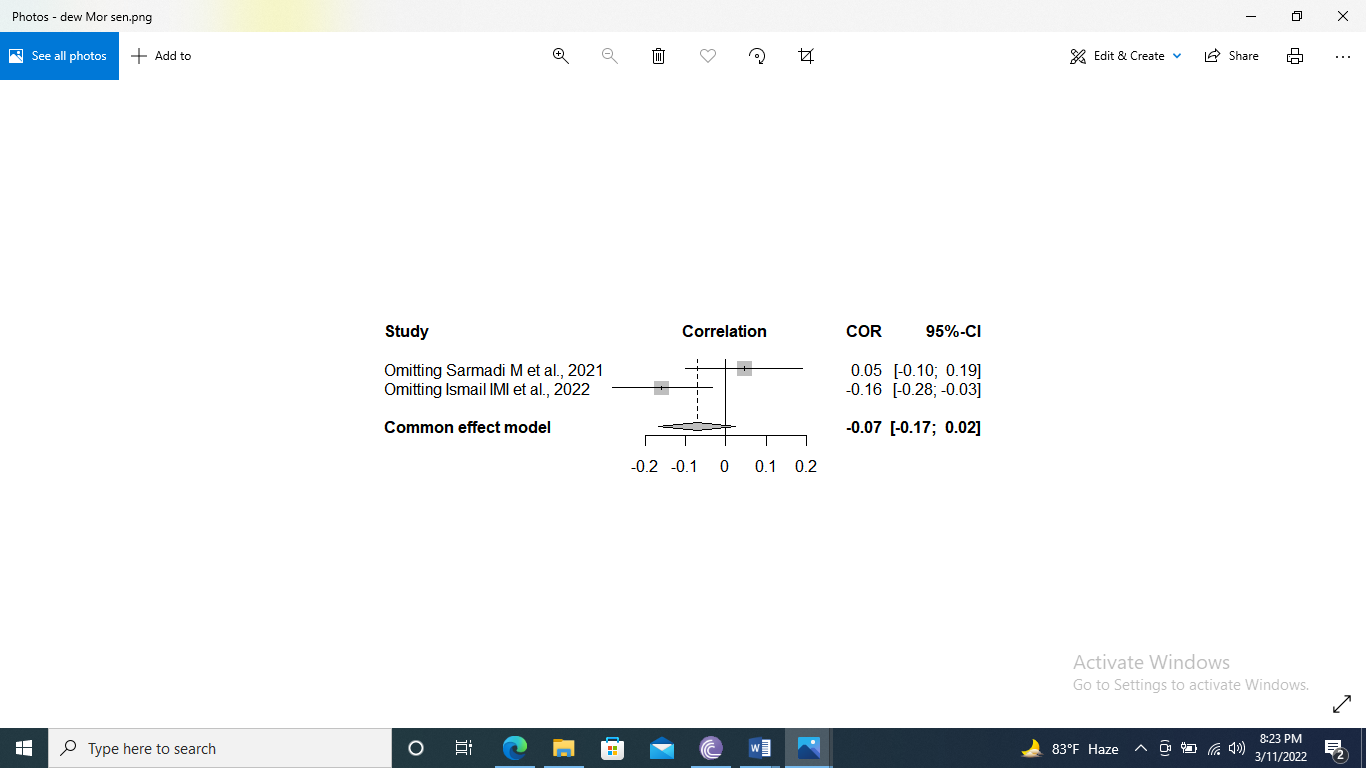


**Fig. S52**: Forest plot of sensitivity analysis of COVID-19 deaths and dew point.


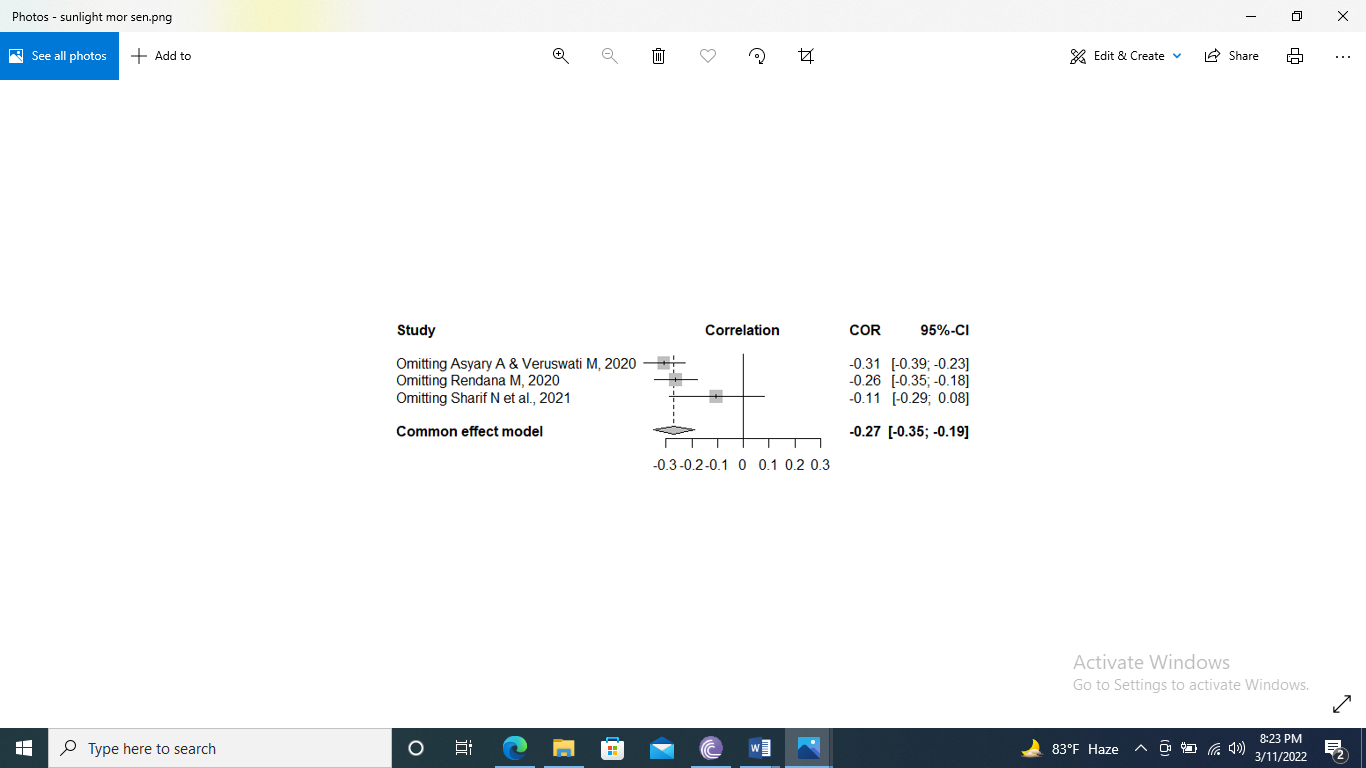


**Fig. S53**: Forest plot of sensitivity analysis of COVID-19 deaths and sunlight.

**Appendix A:**

**Search Strategy:**

**PubMed**

((((((coronavirus[MeSH Terms]) OR covid-19[MeSH Terms]) OR SARS-CoV-2[MeSH Terms]) OR Severe Acute Respiratory Syndrome related coronavirus[MeSH Terms])) AND correlation) AND (((meteorological variable) OR climate variable) OR meteorological factors)

**Scopus**

("coronavirus" OR "COVID-19" OR "novel coronavirus" OR "SARS-CoV-2" OR "2019-nCoV" OR "Severe Acute Respiratory Syndrome related coronavirus") AND ("correlation") AND ("meteorological variable" OR “climate variable” OR “meteorological factors”)

**Science direct**

("coronavirus" OR "COVID-19" OR "novel coronavirus" OR "SARS-CoV-2" OR "Severe Acute Respiratory Syndrome related coronavirus") AND ("correlation") AND ("meteorological variable" OR “climate variable” OR “meteorological factors”)

**Ovid(Medline)**

1 exp Coronavirus Infections/ or exp Coronavirus/

2 exp COVID-19/

3 exp SARS-CoV-2/ or exp Coronavirus/ or exp Severe Acute Respiratory Syndrome/ or exp SARS Virus/

4 correlation.mp.

5 exp Meteorological Concepts/ or exp Seasons/ or exp Air Pollutants/ or exp Weather/ or exp Temperature/ or exp Air Pollution/ or exp Environmental Monitoring/

6 exp Climate Change/ or exp Climate/

7 1 or 2 or 3

8 5 or 6

9 7 and 8

**Ovid (embase)**

1 exp Coronavirus infection/ or exp Coronavirus/ or exp severe acute respiratory syndrome/ or exp coronavirus disease 2019/

2 covid-19.mp.

3 SARS-CoV-2.mp. or exp Severe acute respiratory syndrome coronavirus 2/

4 Severe Acute Respiratory Syndrome related coronavirus.mp. or exp SARS-related coronavirus/

5 correlation.mp.

6 exp meteorology/ or exp humidity/ or exp climate change/ or meteorological variable.mp. or exp seasonal variation/ or exp meteorological phenomena/ or exp air pollution/

7 exp climate/ or exp rain/ or climate variable.mp. or exp climate change/

8 meteorological factors.mp.

9 1 or 2 or 3 or 4

10 6 or 7 or 8

11 9 and 10

**Table S1**: Quality Assessment of all the included articles.

| Author | Year | Q1 | Q2 | Q3 | Q4 | Q5 | Q6 | Q7 | Q8 | Total | Decision |
| --- | --- | --- | --- | --- | --- | --- | --- | --- | --- | --- | --- |
| Asyary A & Veruswati M | 2020 | 1 | 1 | 1 | 1 | 0 | 0 | 1 | 1 | 6 | High |
| Menebo MM | 2020 | 1 | 1 | 1 | 0 | 0 | 0 | 1 | 1 | 5 | Medium |
| Dogan B et al. | 2020 | 1 | 1 | 1 | 1 | 1 | 1 | 1 | 1 | 8 | High |
| Zoran MA et al. | 2022 | 1 | 1 | 1 | 1 | 1 | 1 | 1 | 1 | 8 | High |
| Tosepu R et al. | 2020 | 1 | 1 | 1 | 0 | 0 | 0 | 1 | 1 | 5 | Medium |
| Bashir MF et al. | 2020 | 1 | 1 | 1 | 0 | 1 | 0 | 1 | 1 | 6 | High |
| Zhang Z et al. | 2020 | 1 | 1 | 0 | 1 | 1 | 1 | 1 | 1 | 7 | High |
| Rosario DKA et al. | 2020 | 1 | 1 | 1 | 1 | 0 | 0 | 1 | 1 | 6 | High |
| Ishii T et al. | 2021 | 1 | 1 | 1 | 1 | 0 | 0 | 1 | 1 | 6 | High |
| MEO SA et al. | 2020 | 1 | 1 | 1 | 1 | 0 | 0 | 1 | 1 | 6 | High |
| Islam ARMT et al. | 2020 | 1 | 1 | 1 | 1 | 0 | 0 | 1 | 1 | 6 | High |
| Gautam S et al. | 2021 | 1 | 1 | 1 | 1 | 1 | 0 | 1 | 1 | 7 | High |
| Shahzad K et al. | 2020 | 1 | 1 | 1 | 1 | 1 | 0 | 1 | 1 | 7 | High |
| Lasisi TT & Eluwole KK | 2020 | 1 | 1 | 1 | 1 | 0 | 0 | 1 | 1 | 6 | High |
| Sahoo MM | 2020 | 1 | 1 | 1 | 1 | 1 | 1 | 1 | 1 | 8 | High |
| Sarmadi M et al. | 2021 | 1 | 1 | 1 | 0 | 1 | 1 | 1 | 1 | 7 | High |
| Suhaimi NF et al. | 2020 | 1 | 1 | 1 | 0 | 0 | 0 | 1 | 1 | 5 | Medium |
| Abdelhafez E et al. | 2021 | 1 | 1 | 1 | 0 | 1 | 1 | 1 | 1 | 7 | High |
| Gonçalves J et al. | 2021 | 1 | 1 |  | 1 | 1 | 0 | 1 | 1 | 6 | High |
| MEO SA et al. | 2020 | 1 | 1 | 0 | 1 | 0 | 0 | 1 | 1 | 5 | Medium |
| Bolaño-Ortiz TR et al. | 2020 | 1 | 1 | 1 | 0 | 1 | 1 | 1 | 1 | 7 | High |
| Zheng Z et al. | 2021 | 1 | 1 | 1 | 1 | 1 | 1 | 1 | 1 | 8 | High |
| Abraham J e al. | 2021 | 1 | 1 | 1 | 1 | 1 | 1 | 1 | 1 | 8 | High |
| Rendana M | 2020 | 1 | 1 | 1 | 1 | 0 | 0 | 1 | 1 | 6 | High |
| Pani SK et al. | 2020 | 1 | 1 | 1 | 1 | 0 | 0 | 1 | 1 | 6 | High |
| Zoran MA et al. | 2020 | 1 | 1 | 1 | 1 | 1 | 0 | 1 | 1 | 7 | High |
| Alkhowailed M et al. | 2020 | 1 | 1 | 1 | 1 | 0 | 0 | 1 | 1 | 6 | High |
| Ismail IMI et al. | 2022 | 1 | 1 | 1 | 1 | 1 | 1 | 1 | 1 | 8 | High |
| Kumar G & Kumar RR | 2020 | 1 | 1 | 1 | 1 | 0 | 0 | 1 | 1 | 6 | High |
| Wang Q et al. | 2021 | 1 | 1 | 1 | 1 | 1 | 0 | 1 | 1 | 7 | High |
| Sangkham S et al. | 2021 | 1 | 1 | 1 | 1 | 1 | 0 | 1 | 1 | 7 | High |
| Werner PA et al. | 2021 | 1 | 1 | 1 | 1 | 0 | 0 | 1 | 1 | 6 | High |
| Sharif N et al. | 2021 | 1 | 1 | 1 | 1 | 0 | 0 | 1 | 1 | 6 | High |
| Sharif N and Dey SK | 2020 | 1 | 1 | 1 | 1 | 0 | 0 | 1 | 1 | 6 | High |
| Mofijur M et al. | 2020 | 1 | 1 | 1 | 1 | 1 | 1 | 1 | 1 | 8 | High |
| Chang SA et al. | 2021 | 1 | 1 | 1 | 1 | 0 | 0 | 1 | 1 | 6 | High |
| Bilal et al. | 2021 | 1 | 1 | 0 | 1 | 1 | 1 | 1 | 1 | 7 | High |
| Gautam S et al. | 2021 | 1 | 1 | 1 | 1 | 1 | 1 | 1 | 1 | 8 | High |

Q1: Were the criteria for inclusion in the sample clearly defined?

Q2: Were the study subjects and the setting described in detail?

Q3: Was the exposure measured in a valid and reliable way?

Q4: Were objective, standard criteria used for measurement of the condition?

Q5: Were confounding factors identified?

Q6: Were strategies to deal with confounding factors stated?

Q7: Were the outcomes measured in a valid and reliable way? And

Q8: Was appropriate statistical analysis used?
